# Mechanical adaptation of brachiopod shells via hydration-induced structural changes

**DOI:** 10.1038/s41467-021-25613-4

**Published:** 2021-09-10

**Authors:** Johannes Ihli, Anna S. Schenk, Sabine Rosenfeldt, Klaus Wakonig, Mirko Holler, Giuseppe Falini, Luca Pasquini, Eugénia Delacou, Jim Buckman, Thomas S. Glen, Thomas Kress, Esther H. R. Tsai, David G. Reid, Melinda J. Duer, Maggie Cusack, Fabio Nudelman

**Affiliations:** 1 Photon Science Division, Paul Scherrer Institut, Villigen PSI, Switzerland; 2 Department of Chemistry, Faculty of Biology, Chemistry & Earth Sciences, University of Bayreuth, and Bavarian Polymer Institute, Universitaetsstrasse 30, Bayreuth, Germany; 3 ETH and University of Zürich, Institute for Biomedical Engineering, 8093, Zürich, Switzerland; 4 Dipartimento di Chimica “Giacomo Ciamician”, Alma Mater Studiorum Università di Bologna, via F. Selmi 2, Bologna, Italy; 5 Department of Physics and Astronomy, University of Bologna, viale Berti-Pichat 6/2, Bologna, Italy; 6 School of Chemistry, the University of Edinburgh, Joseph Black Building, Edinburgh, UK; 7 Institute of GeoEnergy Engineering, School of Energy, Geoscience, Infrastructure and Society, Heriot-Watt University, Riccarton, Edinburgh, UK; 8 School of Physics and Astronomy, University of Edinburgh, Edinburgh, UK; 9 Department of Chemistry, University of Cambridge, Cambridge, UK; 10 Center for Functional Nanomaterials, Brookhaven National Laboratory, Upton, NY USA; 11 Munster Technological University, Bishopstown, Cork, T12 P928 & Tralee, Kerry, Cork, Ireland

**Keywords:** Biomaterials, Biomineralization, Imaging techniques

## Abstract

The function-optimized properties of biominerals arise from the hierarchical organization of primary building blocks. Alteration of properties in response to environmental stresses generally involves time-intensive processes of resorption and reprecipitation of mineral in the underlying organic scaffold. Here, we report that the load-bearing shells of the brachiopod *Discinisca tenuis* are an exception to this process. These shells can dynamically modulate their mechanical properties in response to a change in environment, switching from hard and stiff when dry to malleable when hydrated within minutes. Using ptychographic X-ray tomography, electron microscopy and spectroscopy, we describe their hierarchical structure and composition as a function of hydration to understand the structural motifs that generate this adaptability. Key is a complementary set of structural modifications, starting with the swelling of an organic matrix on the micron level via nanocrystal reorganization and ending in an intercalation process on the molecular level in response to hydration.

## Abstract (web summary)

Bioinspired materials require an understanding of how biomaterials achieve the materials properties. Here, the authors report on the load-bearing shell of *Discinisca tenuis* and explore how hydration changes the dry shell from hard and stiff to soft and flexible within minutes by reorganisation caused by organic matrix swelling.

## Introduction

For hundreds of millions of years, nature has evolved a large assortment of organic–inorganic hybrid materials such as bone, teeth, and shells. Each of these biominerals exhibits material properties that have been optimized to aid a particular function, such as navigation, protection, or mechanical support^1,2^. These properties arise from a three-dimensional multi-scale organization of the biomineral’s primary building blocks, e.g., inorganic nanocrystals, specialized proteins, and polysaccharides, from the molecular to the millimeter scale^3–5^. Biominerals with load-bearing functions are optimized, in particular, with respect to their mechanical properties, so as to provide sufficient stiffness to support the typical mechanical loads in the biomineral’s environment and enough toughness to resist crack propagation^3^. This optimization is achieved, first, by incorporating organic biopolymers within the inorganic phase, which increases the toughness of the inherently brittle mineral^6^, and second by organizing the basic building blocks of the tissue into higher-order structures^7^. This hierarchical organization creates a large number of internal interfaces that help to avoid crack propagation and significantly increases fracture toughness. A further advantage of a hierarchical structure is that it endows the organism with an additional level of constructional control, where the basic building blocks can be assembled into different structural motifs of different mechanical properties^8^. Altering the material properties of the biomineral in response to environmental stresses, generally requires active restructuring by remodeling by the organism. A time- and energy-consuming process that involves the resorption of the existing biomineral, followed by the precipitation of new tissue with a different structure and composition^9,10^.

In this paper, we report that the load-bearing shells of the brachiopod *Discinisca tenuis*^11^ are able to dynamically modulate their mechanical properties in response to a change in the environment without the need for remodeling via resorption and regeneration of the tissue, i.e., they switch from hard and stiff when dry to malleable when hydrated within minutes. Importantly, when hydrated the shell can freely bend to the point that it can be folded in two without fracturing.

The effects that water and organic matrix hydration degree have on the mechanical properties of biominerals are well recognized^12^. Water, as a component of most biominerals, is known to increase the flexibility of materials such as bone, teeth, and shells. Modulation of hardness and elastic modulus/flexibility^13^ by passive control of the water content of the tissue has been suggested to occur in non-mineralized insect cuticle^14^ and in mineralized crustacean cuticles, both of which contain organic matrices composed of chitin and proteins^15,16^. In these cases, the changes in mechanical properties are due to the plasticizing role of water and do not involve major changes in the structure of the tissue^12,16^. However, none of these aforementioned mineralized tissues exhibit flexibility that is comparable to that of the mineralized *D. tenuis* shell when in their natural, hydrated state.

We hypothesized that the extreme flexibility of the hydrated *D. tenuis* shell cannot be accounted for solely by the plasticizing effect of water as in these other examples. Rather, such reversibility between stiff and flexible as a function of hydration must have its origins in the structure of the *D. tenuis* shell with water promoting structural changes at different hierarchical levels. The mechanisms that underpin these changes in mechanical properties as a function of hydration are unknown. Chemically controllable material properties and the causal structural motives are of significant interest in the design of stimuli-responsive synthetic materials^17^. As such, there is imperative to determine how hydration alters the structure of the *D. tenuis* shell, and how these changes facilitate the modulation in mechanical properties.

Using a combination of ptychographic X-ray tomography, electron microscopy, small- and wide-angle X-ray scattering, solid-state nuclear magnetic resonance spectroscopy, and mechanical testing, we characterized the shell’s hierarchical structure and composition as a function of hydration covering the micro- and nanoscales and provide an insight into molecular changes. We demonstrate that water absorption by the shell induces a complementary set of structural modifications, starting with the swelling of an organic matrix on the micron level, via nanocrystal reorganization and restructuring, and ending in the intercalation of water between the organic framework and the mineral on the molecular level. In combination, we propose that these changes endow the shell with its mechanical adaptability. We envisage that these observations will aid/ inspire the design of novel synthetic materials with properties that can be modulated in real-time.

## Results

### Global compositional analysis

**Fig. 1:**
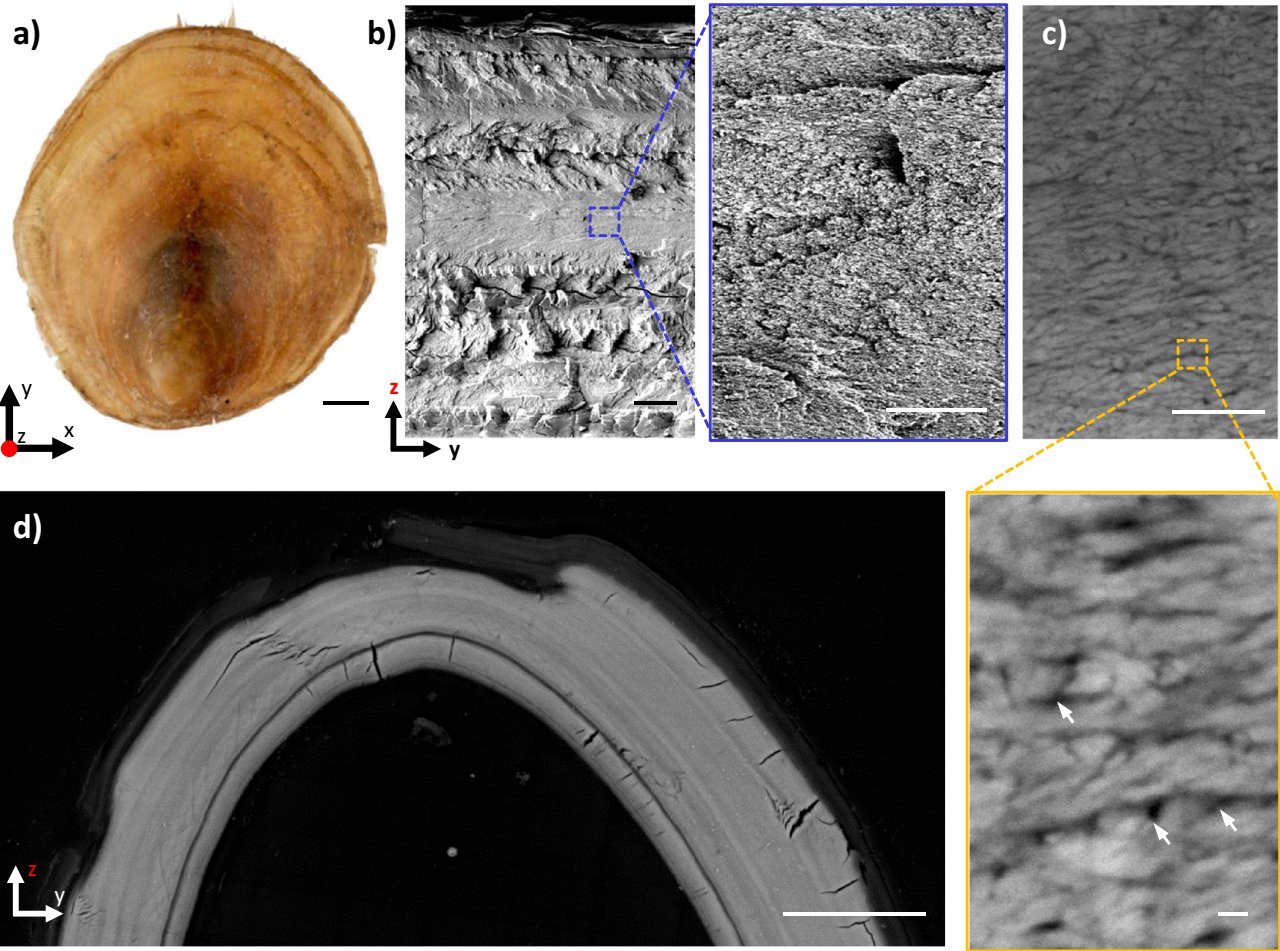
Hierarchical structure of a brachiopod *Discinisca tenuis* shell. **a** Top-down optical micrograph of a dry brachiopod *Discinisca tenuis* shell. Scale bar is 2 mm. **b** Cross-sectional scanning electron microscopy images of the dry shell at increasing magnification. Left: low magnification image showing the cross-section across the *z*-axis. Right: high magnification of the area marked by the dotted square. Scale bars are 20 µm (left), 2 µm (right). **c** High-angle annular dark-field scanning-transmission electron microscopy (HAADF-STEM) images of a thin section from a dry shell. Scale bars are 200 and 20 nm. White arrows point to the organic matrix component (dark areas) surrounding the mineral (bright areas). **d** Cross-sectional electron micrograph acquired with backscattered electrons (BSE) of a fully hydrated shell thin section folded in two. Scale bar 50 µm. Source data for this figure are available at the University of Edinburgh DataShare, data identifier 10.7488/ds/3056^67^.

The shells of *D. tenuis* (Fig. 1a) are an organic-inorganic composite material, where the mineral phase constitutes about 68 wt% of the dry shell^11^. The mineral phase is composed predominantly of carbonate-substituted fluorapatite crystals in the form of francolite^18^ (Supplementary Fig. 1) with minor contributions of amorphous calcium phosphate, octacalcium phosphate, and tricalcium phosphate^19^. The remaining ~32 wt% of the shell consist of various organic fractions, of which chitin, glycosaminoglycans, and proteins make up the dominant portion^11,19–21^. While these shells do not exhibit discernible structural motifs at the micron scale (Fig. 1b), high-angle annular dark-field scanning transmission electron microscopy (HAADF-STEM) has shown that they are hierarchically structured consisting of a laminated brick-work-like structure. This structure is arranged normal to the shell height, where francolite crystals (Fig. 1c, bright objects) are enwrapped by a network of chitin and proteins (Fig. 1c, dark regions).

Importantly, these shells which are hard and brittle when dry, significantly increase in flexibility upon hydration to the point where they can be folded in half without fracturing as seen in Fig. 1d and supplementary Movie 1. This process is reversible so that shells can be cycled multiple times through hard/brittle and soft/flexible by dehydrating/rehydrating.

Thermogravimetric analysis (TGA) was used to determine the water content of an atmospherically dry shell stored in the air as compared to a fully hydrated shell after immersion in H_2_O for 24 h (Supplementary Fig. 2). The dry shell displayed a gradual water weight loss of 5% from ambient temperature to 200 °C. The hydrated shell exhibits a water weight loss of 24% across the same temperature range. Notably, in the hydrated shell, these losses occur in two distinct steps, the first at 60 °C, corresponding to the loss of physisorbed water accounting for ~18 wt% H_2_O and a more gradual secondary loss between ~100 and 200 °C (~6 wt% H_2_O). Further weight loss steps, observed for both the dry and the hydrated shell, occurring above 287 and 400 °C, are related to the pyrolysis of the organic components^22,23^, while decarbonation occurs above 700 °C and results in the formation of fluorapatite (Supplementary Fig. 3).

### Mechanical behavior characterization

Depth-sensing nanoindentation was used to determine the mechanical properties of the shell as a function of the degree of hydration. Due to practical and geometrical constraints, i.e., the brachiopod’s shell is curved, has an irregular surface and a thickness of 50–500 µm that is both shell- and location-dependent, depth-dependent dynamic nanoindentation measurements were performed on shell cross-sections. These cross-sections expose the laminated structure (Fig. 1b), i.e., the indentation direction is in the plane of the laminae. Measurements were performed on atmospherically dry and fully hydrated shells on the same sample, at the center of the cross-section or shell diameter.

**Fig. 2:**
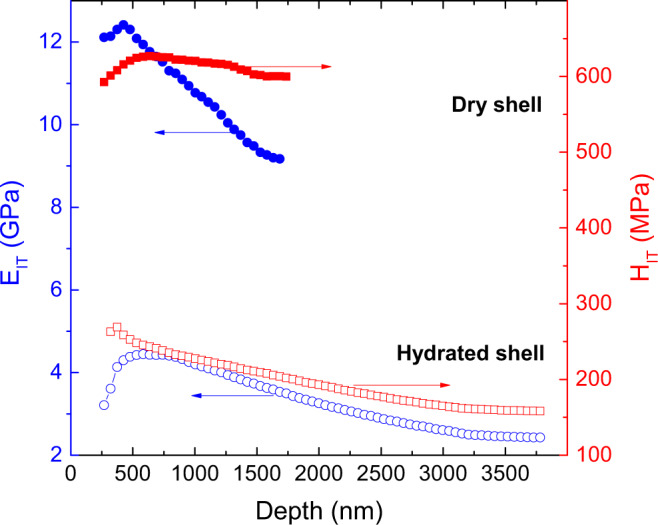
Depth-dependent dynamic nanoindentation measurements of *D. tenuis* brachiopod shell samples at different hydration levels. Plotted is the dependence of shell hardness *H*_IT_ (red, right) and Young’s modulus *E*_IT_ (blue, left) as a function of indentation depth for an atmospherically dry shell sample (top, filled symbols) and a fully hydrated shell sample (bottom, open symbols). Measurements were conducted in continuous stiffness mode up to a maximum force of 30 mN. Shown is the average over eight indentation measurements.

Depth-sensing nanoindentation measurements show that both Young’s modulus (*E*_IT_) and hardness (*H*_IT_) drop drastically when the shell becomes hydrated (Fig. 2). At the maximum tested load of 30 mN, *E*_IT_ is about 26% of the dry value and *H*_IT_ shows a similar reduction down to 22% (Supplementary Table 1). The greater deformability of the hydrated sections under the same applied load is demonstrated also by the increase of the maximum indentation depth from ~1.5 to ~3.5 µm at 30 mN (Supplementary Fig. 4), as well as by the larger residual indentation imprint (Supplementary Fig. 5).

### Micron-scale characterization

Ptychographic X-ray computed tomography (PXCT) was used to characterize the shell structure at increasing levels of hydration on the micron and sub-micron levels. PXCT provided quantitative electron density tomograms (*n*_e_Å^−^^3^)^24–26^, with a half-period spatial resolution of roughly 85 nm (Supplementary Fig. 6). These tomograms allow a structural evaluation of the shell and provide information such as local variations in swelling behavior and the hydration degree of specific components in the sample, e.g., organic- and mineral-rich regions.

Sample cylinders, greater than 15 µm in diameter, were extracted along the shell width (*z-*axis in Fig. 1b) and prepared using a nitrogen-cooled micro-lath (Supplementary Movie 2)^27^. These cylinders were then either vacuum-dried or incubated at 70 % or 100 % relative humidity (RH) for 36 h^28,29^, resulting in samples of increasing hydration level. Lastly, samples were flash-frozen in liquid nitrogen and analyzed using cryo-PXCT at −180 °C.

**Fig. 3:**
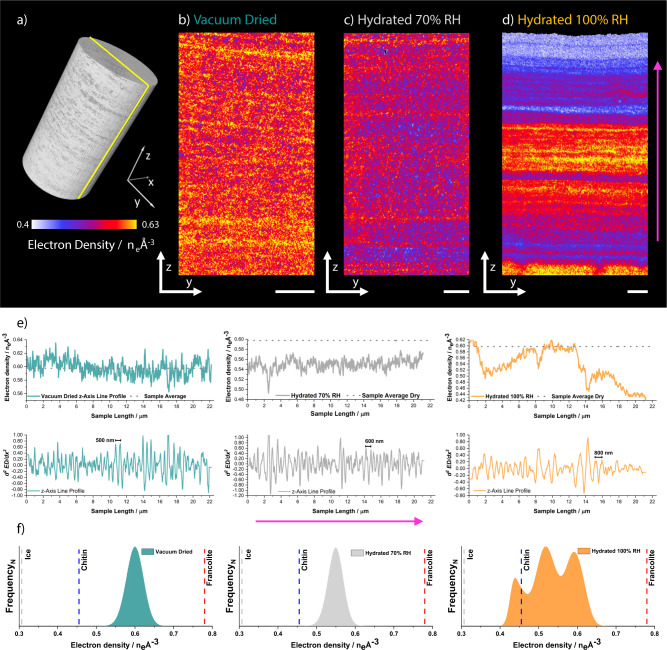
Electron density tomograms of *D. tenuis* brachiopod shell samples at increasing hydration level. **a** Example volume rendering of the imaged, cylindrical, sample pillars. Sagittal cut slices through the center of **b** a vacuum dried sample, **c** a sample incubated at 70% RH, and **d** a sample incubated at 100% RH. The cutting plane is represented by the yellow line shown in (**a**). Scale bars are 2 µm. Common to all cuts is a single color scale ranging from white to yellow representative of electron density values. Shown in **e** are sample plane averaged electron density line profiles normal to the laminae structure (pink arrow) alongside secondary derivatives highlighting major fluctuations in electron density. Sample corresponding frequency normalized (*N*) electron density histograms are shown in (**f**). Further provided in **f** are the theoretical electron density values of the shell main components: francolite 0.78 n_e_Å^−^^3^, high molecular weight polysaccharides approximated using chitin ~0.46 n_e_Å^−3^, and low-density amorphous ice, 0.31 n_e_Å^−3^. PCXT measurements were conducted under cryogenic conditions. The voxel size of all tomograms is (38.8 nm)^3^. Source data for this figure are available at the University of Edinburgh DataShare, data identifier 10.7488/ds/3056^67^.

PXCT-derived electron density tomograms are shown in Fig. 3. As the vacuum-dried sample can be described as a two-component system of mineral and organic fractions, each with approximately known electron densities of 0.78 n_e_Å^−^^3^ (francolite) and 0.46 n_e_Å^−3^ (chitin), the measured electron densities can be used to determine the shell’s composition globally and locally in consideration of partial volume effects^24,30^. Partial volume effects refer to the occupation of a volume element, e.g., a voxel, by multiple components, leading to a fractional occupancy-related electron density. For example, the average electron density of the vacuum-dried sample of 0.59 n_e_Å^−3^, suggests that the vacuum-dried shell consists of ~58 vol% organics or roughly 33 wt%, as previously reported^11,31^. Moreover, using the vacuum-dried shell as a compositional reference point, the average water content in the hydrated samples can be estimated, i.e., observable changes in electron density are attributed to the incorporation of water into the shell structure. In detail, whereas the shell sample stored at 70% RH contains roughly 17 vol% water or ~4 wt%, the sample incubated at 100% RH possesses up to 50 vol% water or ~12 wt%. Although the hydration level of the sample stored at 70% RH is comparable with that of the atmospherically dry sample shown in Fig. 1c, a lower hydration level was measured for the 100% RH sample by PCXT when compared to the shell sample fully immersed in water shown in Supplementary Fig. 2. This discrepancy is a result of the sample cylinder hydration process, which was used to avoid structural alteration of the shell during the freezing process.

Figure 3a provides an example volume rendering of one of the imaged sample cylinders. Sagittal slices through the acquired electron density tomograms are presented in Fig. 3b–d and Supplementary Movies 3–5. These slices reveal a progressively more defined laminar structure of alternating high electron density, mineral-rich layers and low electron density, organic-rich layers normal to the shell height from the dry to the fully hydrated sample. To quantify the separation of these layers and emphasize local variations in their thickness and composition, layer-averaged electron density profiles normal to the laminae structure were calculated (Fig. 3e). Visible in these profiles is a continued expansion of the organic-rich layer thickness from ~160 nm in the dry sample to ~180 nm in the partially hydrated sample (70 RH) to ~340 nm in the fully hydrated sample (100 RH). The mineral-rich layers also appear to expand, although to a much lesser extent. From ~90 nm in the dry sample to ~110 nm in the partially and fully hydrated samples. These data suggest the existence of two local hydration environments, associated with either the mineral-rich or the organic-rich layers, each possessing a distinct hydration capacity.

The corresponding electron density histograms of the tomograms are supportive of this interpretation (Fig. 3f). With increasing hydration, an evolution of the Gaussian distribution centered at 0.59 n_e_Å^−3^ is visible. Partial hydration results in a shift of the Gaussian distribution towards an electron density center at 0.54 n_e_Å^−^^3^. This shift, while retaining the Gaussian distribution, suggests a near-uniform water uptake throughout the shell, i.e., including the francolite nanoparticle enwrapping organics. Further hydration leads to the development of a broad and asymmetric electron density distribution with 3 main peaks: centered at 0.42, 0.51, and 0.59 n_e_Å^−3^. While the retained peak at 0.59 n_e_Å^−3^ and the newly emergent peak at 0.42 n_e_Å^−3^ can reasonably be assigned to mineral-rich domains and fully hydrated organic-rich domains respectively, the persistence and dominance of the intermediate peak are intriguing. It is indicative of either a not yet fully completed hydration process or the presence of not one but two organic-rich layer structures in the shell each with different hydration capacities.

To investigate the existence of such a variety in structure and to establish a correlation between the degree of hydration and volume expansion of the organic-rich layers, we remapped the electron density tomogram of the fully hydrated sample to percent water weight. Subsequently, we characterized the organic-rich layers in this tomogram, which revealed that within a single layer, hydration is rather uniform. However, hydration can and does vary across layers. Visible in the tomogram is a zonation in hydration along with the shell height. Organic-rich layers in close proximity to the shell exterior and extending microns in height, compartmentalize up to ~70 vol% of water or ~24 wt%. Organic-rich layer toward the shell interior adopts a hydration level of around 8 wt% H_2_O. This zonation is in agreement with the variety in organic-rich layer structure detected in the histogram (see Methods and Supplementary Fig. 7).

### Nanometer-scale characterization

As PXCT measurements are limited in spatial-resolution, e.g., a single voxel is occupied by multiple organic matrix-coated francolite crystals (Fig. 1c), we used backscattered electron-scanning electron microscopy (BSE-SEM), offering a higher resolving power, to confirm and expand on these observations. Further BSE-SEM allowed us to resolve the shell’s fine structure and probe the hydration behavior on the nanoscale. Cross-sections of a fully hydrated (fixed in 4% formaldehyde) and a dry shell stored in the air were prepared through a series of dehydration, critical-point drying, resin embedding, and mechanical polishing (details in Methods).

**Fig. 4:**
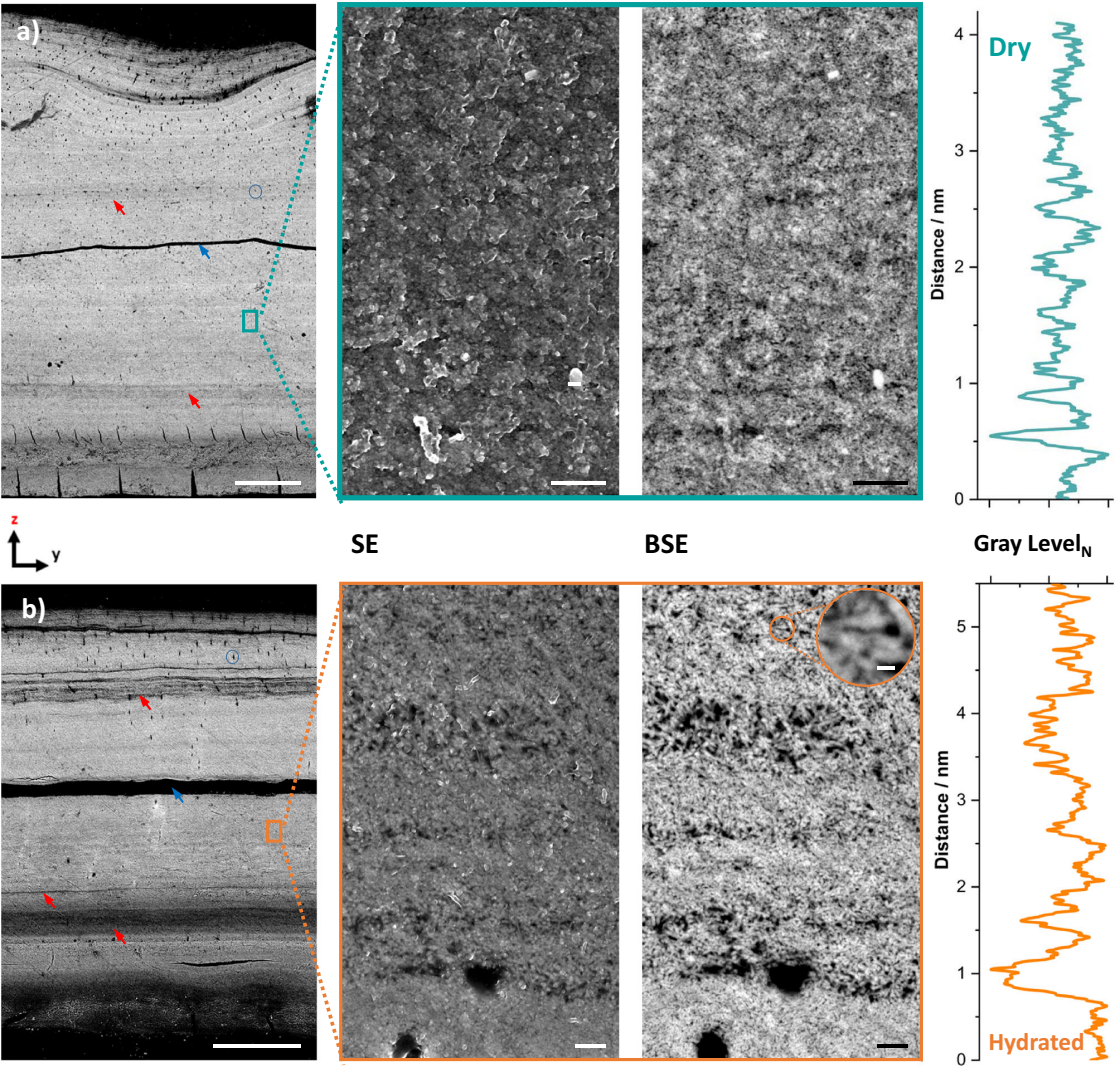
Scanning electron microscopy of polished shell samples. Shown in the left panels are SEM-BSE cross-section micrographs of **a** a dry shell stored in air and **b** a fully hydrated shell fixed with 4% formaldehyde. Blue arrows highlight fracture lines incurred during sample preparation. Red arrows are used to point out example areas of high organic content. Blue circles are used to indicate pores in the shell. Scale bars are 20 µm. Provided in the central panels are images at a higher magnification acquired either with secondary electrons (SE) to stress variations in morphology and surface topography or acquired with backscattered electrons (BSE) used to highlight compositional/elemental contrast. Scale bars are 200 nm. The inset highlights the francolite bundle dimension. The scale bar is 50 nm. In the far right panels are average line profiles of the grayscale within the colored boxes in (**a**) and (**b**), normal to the laminae direction. Source data for this figure are available at the University of Edinburgh DataShare, data identifier 10.7488/ds/3056^67^.

Overview micrographs confirm both the presence of organic-rich layers of varying thickness and their volume expansion upon hydration (Fig. 4a, b). The volume expansion is suggested to occur through the uptake of physisorbed water. In addition, given the available cross-sectional view of the entire shell height in these micrographs, it is evident that major organic-rich layers are predominantly located toward both the outer and inner surfaces. Equally visible in these micrographs is a sparse network of transport channels, <400 nm in diameter^11^, throughout the entire shell, exhibiting a tortuous structure running dominantly normal to the laminae structure. See also Supplementary Movies 3 and 4 and complementary small-angle X-ray scattering measurements shown in Supplementary Fig. 8. In addition, PXCT data show that these pores change in electron density from ~0.05 to 0.2 n_e_Å^−3^ in the vacuum-dried sample to ~0.3–0.35 n_e_Å^−3^ in the hydrated sample (Supplementary Fig. 9). This change is in agreement with the pores carrying water in the hydrated state of the shell; for comparison, the electron density of amorphous ice is 0.31 n_e_Å^−3^

Selected-area high magnification SEM images (Fig. 4b) probing the nanoscopic level, not only confirm the swelling of organic components at this level but additionally discloses another feature of the shell’s organization. Throughout the shell, francolite crystals appear to be organized in individual, rod-shaped bundles, roughly 25 nm in diameter and 100 nm in length (also seen in Fig. 1c and Supplementary Fig. 8). These bundles are found within both mineral- and organic-rich layers, and appear to expand in volume upon hydration, suggesting that they are composed of francolite nanocrystals and organic matrix elements. PXCT measurements confirm this assessment, as the highest voxel-level recorded electron density is still well below that of francolite, confirming that each bundle possesses a minimum organic content of 15 vol%. The nanocrystals within a bundle are asymmetric in shape with a short axis, 3–6 nm in diameter, and a long axis, 14–19 nm in diameter, according to Rietveld’s refinement of powder diffraction data, (Supplementary Fig. 1). The spatial arrangement of the francolite crystals, evident from Fig. 4b, is that the bundles and the nanocrystals preserve some preferential orientation with their long axis parallel to the laminar direction upon hydration.

### Molecular-scale characterization

**Fig. 5:**
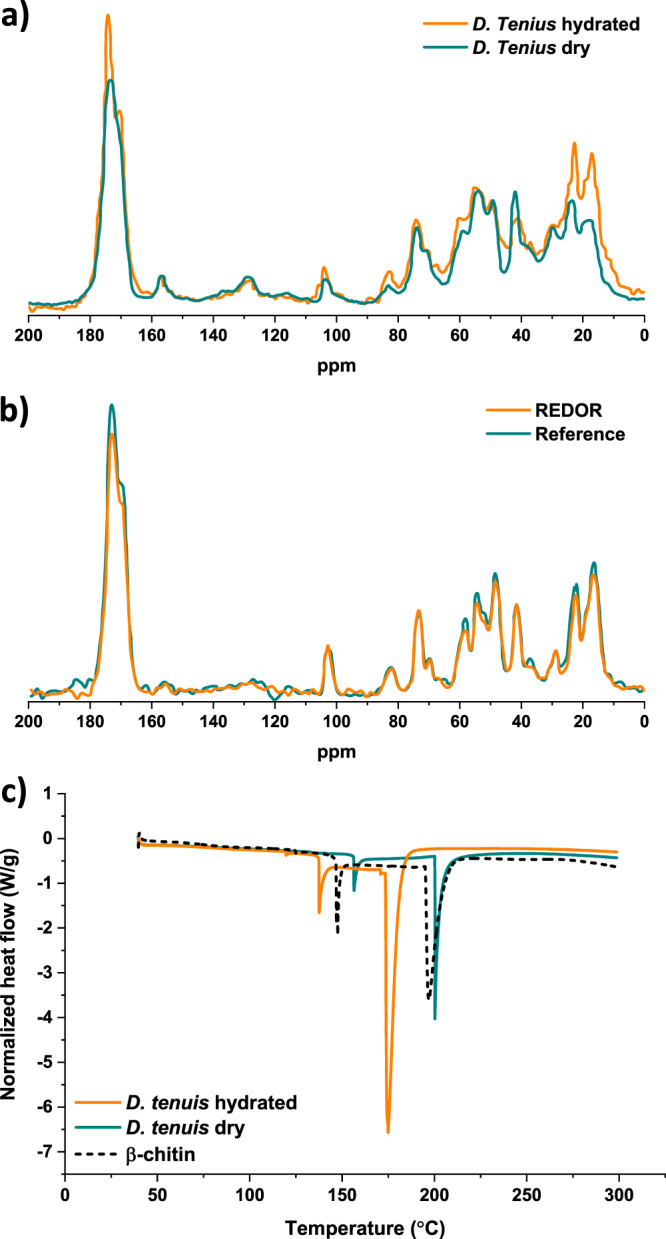
Solid-state nuclear magnetic resonance spectroscopy and differential scanning calorimetry of *D. tenuis* brachiopod shell samples. **a**^13^C Cross-polarization magic angle spinning (CP MAS) NMR spectra of an atmospherically dry and hydrated shell. **b** Rotational-echo double resonance (REDOR) NMR on an atmospherically dry shell, cyan: reference spectrum; orange: REDOR dephasing spectrum. **c** Differential scanning calorimetry measurements of an atmospherically dry and hydrated specimen and of β-chitin extracted from a brachiopod shell.

Solid-state nuclear magnetic resonance (NMR) (ssNMR) spectroscopy and differential scanning calorimetry (DSC) were used to investigate the hydration process of the mineral and of organics surrounding individual francolite crystals on the molecular level. To examine the effect that hydration has on the molecular structure of the organic matrix we collected ^13^C solid-state NMR spectra. Presented in Fig. 5a are ^13^C cross-polarization magic angle spinning NMR (^13^C CP MAS NMR) spectra of a dry and a fully hydrated shell, revealing a hydration-induced sharpening of amide signals (170–180 ppm) to the extent that two amide signals are resolved for the hydrated shell (170.5 and 174.4 ppm) compared to a single broad signal in this region for the dry shell. The chitin N-acetyl methyl group ^13^C signal occurs as expected between 20 and 25 ppm and the N-acetyl C=O ^13^C around 175 ppm. We assign the signals at 22.8 and 174.4 ppm which sharpen with increasing hydration to chitin (or other glycans) N-acetyl groups that become more mobile with hydration of the shell.

To examine the interface between the organic matrix and mineral, ^13^C{^31^P} rotational-echo double resonance (REDOR) NMR spectra were collected to determine which organic functional groups are in closest contact with the phosphate anions of the francolite crystals (Fig. 5b). Signals that have a reduced intensity in the REDOR spectrum compared to the reference spectrum of the dry shell (orange spectrum in Fig. 5b) are indicative of ^13^C sites that are within 0.8–1 nm of ^31^P. An insufficient signal intensity even in the reference spectrum of the hydrated shell sample, due to short T_2_ relaxation times indicate that hydration results in a significant increase in molecular mobility of all organic components of the shell material. Further, signals from methyl groups at 16.9 and 22.8 ppm show a reduction in intensity in the dry REDOR spectrum (orange spectrum in Fig. 5b), along with both amide carbonyl signals, a broad signal at ~185 ppm from carboxylate groups, and a set of signals corresponding in the chemical shift to primary or secondary amine ^13^Cs (50–60 ppm, as labeled in Fig. 5b). Interestingly, there is no reduction in intensity in the REDOR spectrum of signals from chitin/glycan ring carbons, suggesting that these carbons are more than 0.8–1 nm from phosphate. The amide carbonyl signals are from glycan N-acetyl and/or protein–peptide bond carbonyls and the 22.8 ppm signal is from the N-acetyl methyl ^13^C in chitin or other glycans, suggesting that the N-acetyl moieties of chitin/glycan molecules are associated with the mineral.

In summary, the ssNMR data indicate that chitin is organized in layers with its N-acetyl groups facing the mineral where possible, creating inter-layer channels that allow the intercalation of water molecules during hydration. This picture is consistent with the chitin network surrounding the crystals absorbing water. The result is increased mobility of the macromolecular chains and thus flexibility of this particular part of the shell when hydrated.

DSC measurements of entire shells and chitin extracted from *D. tenuis* shells, presented in Fig. 5c, are in general agreement with the suggested hydration process. Not only does the DSC signal of an atmospherically dry shell (5 wt% H_2_O) display two endothermic peaks, one at 156 °C and a second at 200 °C, it also matches the expected stepwise transition of β-chitin dihydrate to its anhydrous form^32^. Importantly, these transitions are recorded at significantly reduced temperatures, 137 and 175 °C (Fig. 5c) in the case of a fully hydrated shell (24 wt% H_2_O). These observations imply that water molecules intercalate between the polysaccharide chains of dihydrate chitin and decrease the inter-chain interactions, making the molecules more mobile. Chemically this can be explained by the preference of the hydroxyl groups in the pyranose ring to make hydrogen bonds with the more mobile solvent molecules rather than with a neighboring sugar residue^33^.

**Fig. 6:**
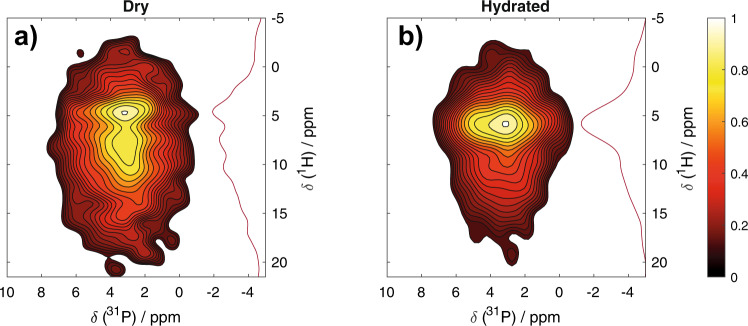
^1^H–^31^P heteronuclear correlation solid-state NMR spectra of *D. tenuis* brachiopod shell samples. Shown are changes in the chemical structure of the atmospherically dry shell (**a**) upon hydration (**b**). The correlation degree follows a normalized color map ranging from red to white (0–1).

Lastly, to characterize the effect that hydration has on the mineral, 2D ^1^H–^31^P heteronuclear correlation solid-state NMR spectra were collected on shell fragments (Fig. 6). Spectra from atmospherically dry shells show multiple distinct ^1^H environments correlated with phosphate ^31^P signals, evident of a well-structured mineral on the molecular length scale. The ^1^H environment near 4.7 ppm is similar to that previously observed for water in the amorphous hydrated surface layer of nanocrystalline hydroxyapatite^34^. The ^1^H signals between ~10 and 15 ppm are from mineral hydrogen phosphate groups with ^1^H chemical shifts that are similar to those found in the amorphous hydrated surface layer of synthetic nanocrystalline hydroxyapatite^35^ and in the hydrated layers of octacalcium phosphate, and in the hydrated calcium hydrogen phosphate phases, monocalcium monohydrate and brushite^36^. There is an additional intriguing ^1^H signal around 7.5 ppm; a similar ^1^H chemical shift was observed in hydroxyapatite samples and has previously been tentatively assigned as hydroxyapatite-associated hydrogen phosphate^36^, and 2D ^1^H–^31^P correlation spectra of synthetic hydroxyapatites contain intensity in this spectral region as well^34^ (Fig. 6a). Upon hydration, the ^1^H spectrum is dominated by a single water signal which has shifted to being centered at 5.9 ppm (Fig. 6b), indicating a significant change in the mineral water environment; the shift to a higher frequency for the water ^1^H resonance suggests the water is in a more strongly hydrogen-bonded environment, such as that in the hydrated layers of OCP or the crystalline water in brushite^36^. These observations suggest that in the hydrated shell, water associated with the mineral is in smaller width channels than in the dry shell. These spectral changes are consistent with a relaxation of strain in the mineral structure upon hydration, as suggested by wide-angle-X-ray-scattering measurements of a shell in its dry and hydrated form (Supplementary Fig. 10) and possibly the result of cracking or partial hydrolysis of mineral crystals and admission of water into the resulting cracks/ hydrolyzed regions.

## Discussion

We show that water absorption causes structural changes in the shell at three levels: (1) At the microscopic level, where organic-rich laminae swell due to the uptake of physisorbed water; (2) at the nanoscopic level, where the organic matrix, surrounding mineral bundles, swells, and (3) at the molecular level, where the chitin network that surrounds the mineral crystals within each bundle become hydrated. This results in a mobility increase of the macromolecular chains of the polysaccharide and in the intercalation of water molecules between chitin and the mineral. These insights allowed us to develop a hypothesis as to how such structural changes translate into the observed mechanical adaptability. Our proposed model and the wider significance of the material properties of the shell are discussed below.

**Fig. 7:**
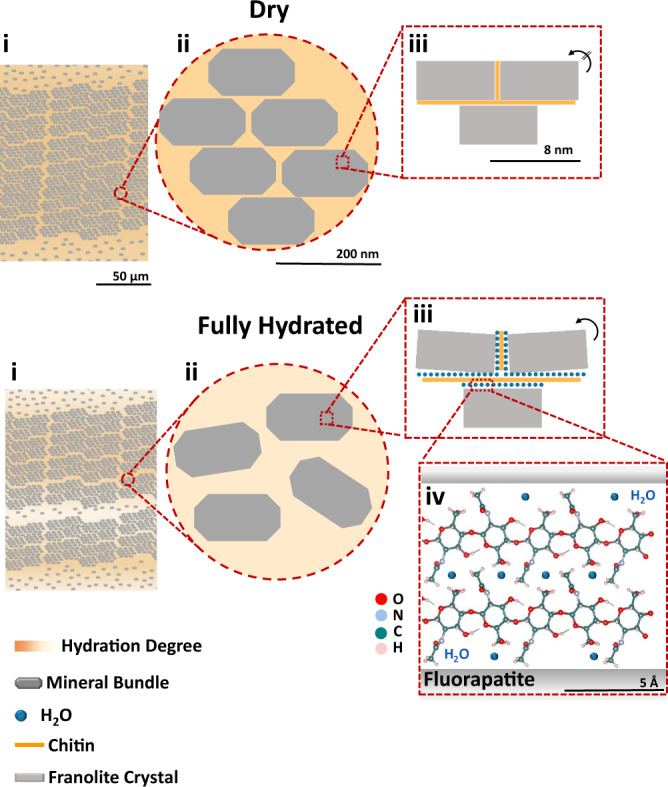
Hydration scheme of a *D. tenuis* brachiopod shell. Schematic of the proposed hydration mechanism across length scales from the micron (i), sub-micron (ii), nano (iii), and molecular level (iv)^33, 68, 69^. In (iii) and (iv), we propose that the intercalation of water between the mineral and chitin enables the mineral units to move more freely when a load is applied.

To explain the structure–property relationship of the shells of *D. tenuis* we propose the following. At the microscopic level, the shell has organic-rich regions which swell when hydrated (Fig. 7i). This results in thicker laminae of low stiffness intercalated with high-stiffness mineral-rich regions, which provides higher flexibility and fracture toughness^37^. At the nano-scale, the mineral-rich regions are composed of francolite crystals assembled into rod-like bundles *ca*. 25 nm in diameter and 100 nm in length surrounded by a network of chitin. This intercalation of two different materials with different elastic moduli—low-stiffness chitin surrounding high-stiffness mineral crystals—is responsible for the inherent high toughness of the shell^38^. We propose that the swelling of the organic components at this level, increasing the disorder in the arrangement of these bundles, facilitates the movement of these structural building blocks when a load is applied (Fig. 7ii). At the molecular level, hydration reduces the stiffness of chitin^39^ by breaking stabilizing hydrogen bonds between the sugar residues^33^, perhaps analogous to how water breaks the inter-peptide bonds in collagen in dentine, decreasing the hardness and stiffness of the latter. It is therefore conceivable that this reduction in the stiffness of chitin, together with the increase in mobility of the polysaccharide side chains, helps to dissipate mechanical stress^40^. In addition, we propose that the intercalation of water molecules between the polysaccharide and the mineral decreases the interaction between these two components so that they can slide/move with respect to each other when under a load (Fig. 7iii). In combination, these structural changes could explain the mechanical adaptability of the shell as a function of the surrounding environment.

In view of the passive, rapid, and repeated adaptability of the shell, a key factor facilitating the hydration process is the efficient transport of water into and out of the shell. As the mechanical properties of the shell change within minutes after immersion in or removal from water, diffusion through the mineral layers is unlikely to be the dominant transport mechanism. As shown in Fig. 4a, b and Supplementary Fig. 9, the shell is permeated by pores that run predominantly normal to the laminae structure. As these pores become filled with water in the hydrated sample (Supplementary Fig. 9) and are interconnected with the protein and chitin networks, as discussed by Williams et al.^11^, it is conceivable that they serve as hydration channels in the mineralized tissue.

In terms of how hydration affects the mechanical properties of the shell, absorption of water causes a reduction in elastic modulus (*E*) and hardness by factors of around four. Considering that hardness is generally proportional to yield stress (*σ*_*y*_), and the shell diameter or thickness (*h*) is nearly unchanged following hydration, this suggests that both the dry and hydrated shell possess roughly the same flexibility (*f*) as defined by Peng and Snyder (2019), *ƒ* = (2/*h*)(*σ*_*y*_/*E*)^13^. Therefore, the increased flexibility observed upon hydration does not originate from a larger decrease in ${{{{{{{\rm{E}}}}}}}}$ compared to *σ*_*y*_.

A decrease in elastic modulus by a factor of four implies that a four times higher elastic strain can be imposed on the hydrated shell compared to the dry one under the same load, possibly explaining why it becomes so easy to bend. Yet, the observed change in hardness, positively correlated with a yield stress, suggests that plastic deformation develops under a four times smaller stress. In other words, when the bending force is increased, the hydrated shell material enters the plastic deformation regime at about four times smaller stress compared to the dry one.

The fact that much larger deformations can be achieved in the hydrated state compared to the dry state can only be explained by considering the presence of some plastic deformation together with a much higher ductility upon hydration (In general, plasticity at smaller stress brings about an enhanced ductility, i.e., the ability to withstand larger plastic deformation without fracture). If the situation were otherwise, it would be possible to achieve the same deformation in the dry shell simply by applying a four times higher force. This is not the case as the dry shell fractures at small strains.

In summary, we suggest: (1) The large deformations in the hydrated shell are never purely elastic but include a certain degree of plasticity; (2) the hydrated shell has a much higher ductility and does not fracture immediately when entering the plastic deformation regime, in contrast to the brittle dry shell; (3) the combined changes of elastic modulus, hardness and ductility with hydration/dehydration determines the macroscopic mechanical behavior of the shell.

It is interesting to compare this behavior with that of other biominerals. Dentine, for example, possesses a similar ratio of organic to mineral content, yet displays a decrease in elastic modulus by a factor of ~1.5, and hardness by a factor of ~3 upon hydration^41^. The crustacean endocuticle, which has a thickness that is more comparable to that of the *D. tenuis* shell (*ca*. 200–300 µm^16^, whereas the shell is 50–500 µm thick), and a more comparable passive change in flexibility with changes in hydration, displays a decrease in stiffness by a factor of ~1.4 upon hydration, while the yield stress changes by a factor of ~4^16^. In the latter, these changes are driven mainly by the interaction of water with the protein that is associated with the chitin fibers^39^, and ingress of water breaking hydrogen bonds between macromolecular chains^33^ or inter-peptide bonds^41^ is a common mechanism by which water increases the flexibility of biominerals. In these cases, water chiefly acts as a plasticizer, increasing the viscoelasticity and plasticity of the organic matrix components^12^ through changes in intermolecular hydrogen bonding in the tissue as discussed above. What sets the *D. tenuis* shells apart from other biominerals is the extent of the flexibility caused by hydration, which is not seen in other mineralized tissues, and the speed of the structural reorganization underlying the change in flexibility. To put it simply, mollucs shells, bone and dentine cannot bend in half without breaking, as the hydrated *D. tenuis* shell can, no matter what their water content.

Considering that bone and dentine have similar amounts of organic and inorganic contents as the *D. tenuis* shell (60–70% inorganic and 30–40% organic), it is clear that the ability of the shell to freely bend is due not only to its high organic content and the plasticizing effect of water. Their differences in mechanical behavior must also be due to how the building blocks of each material are organized. As described above, the arrangement of the francolite nanocrystals into discrete bundles enwrapped by a layer of the organic matrix provides the shell with separate blocks that can move with respect to each other once a load is applied, and the stiffness of chitin and the chitin–mineral interactions are weakened by hydration. Moreover, the mineral itself appears from solid-state NMR measurements, to restructure reversibly upon hydration/dehydration. We speculate that the activation energy for such restructuring comes from the relaxation of crystal strain upon water ingress and the resulting formation of hydrated channels or layers with dimensions of the scale of the water channels and layers in OCP or brushite, for instance. Bone, on the other hand, is made of collagen fibrils with intra- and extra-fibrillar mineral^42–45^. The mineralized collagen fibrils are further arranged into higher-order structures—such as unidirectional ordered fibrils or plywood structures, further arranged into super-structures^3,44^. This hierarchical organization results in a material with high stiffness that resists deformation when under a load. Similarly, other biominerals such as the nacreous layer in molluscs have their mineral building blocks arranged such that they cannot move much with respect to each other when under a bending load. The nacre of bivalves, for example, is made of aragonite tablets that are significantly larger than the crystalline units of the *D. tenuis* shells—500 nm in thickness and 5–15 μm in diameter^46^—and are staggered with respect to each other which readily prevents any deformation in the same scale as reported here for the brachiopod shell. In addition, while these tissues are naturally hydrated, they have not been reported to further uptake significant amounts of water as the *D. tenuis* shell does. As demonstrated, an increase in water content is a prerequisite to increase their flexibility. As for the non-mineralized insect cuticle and the mineralized crustacean cuticles, these tissues are composed of chitin-protein fibers aligned in parallel arrays forming horizontal planes that are stacked vertically with the gradual rotation of the long axis of the fibers around the normal axis of the cuticle, leading to a twisted plywood structure^16,39^. Their hardness and stiffness depend on the stacking density of the chitin–protein layers and on the degree of mineralization^47^. These two structural factors do not change upon hydration and dehydration.

In summary, we conclude that the responsiveness of the mechanical properties of the *D. tenuis* shell to hydration, when compared to other biominerals, is a combination of several factors: (1) the high amount of organic content; (2) the plasticizing role of water on the organic matrix and mineral; (3) the weakening of the interaction between the mineral and chitin upon hydration, allowing the former to move more freely under a load; and (4) the unique hierarchical structure of the shell, with crystals surrounded by a chitin matrix at the nanoscale, and organic-rich layers at the micron scale. While factors (1) and (2) are common among other biominerals, factors (3) and (4) are unique to the *D. tenuis* shell.

To address the lingering question as to why *D. tenuis* brachiopods evolved and currently possess shells of such mechanical adaptability further investigations are needed. Nonetheless, one can draw a parallel between the brachiopod *Lingula anatina*, which also has a phosphatic shell. A certain degree of mechanical adaptability and flexibility in the shells of *L. anatina* was proposed to be needed for the burrowing of the animal into sediment^37^, for its infaunal habitat. It is likely that the mechanical properties of *D. tenuis* shells are similarly suitable to their environment. Large clusters of *D. tenuis* specimens, attached only to each other, inhabit the inter-tidal zone. In such a high-energy environment, with extreme ranges of hydration throughout diurnal tidal cycles, as mimicked in the presented experiments, environment-adapting flexibility could be advantageous to prevent shell damage and therefore could be key to the survival of the animals. Thus, differences between the ecological niches between the two species of phosphatic brachiopods, *D*. *tenuis* and *L. anatina*, or even when compared to calcitic-shelled species, could mean different mechanical requirements for their shells and hence explain the reason *D. tenuis* shells have higher flexibility when hydrated than other brachiopods.

In conclusion, we report on the mechanical behavior of the shells of *D. tenuis*. The former displays passive adaptability among binominerals in mechanical properties as a function of hydration/the environment it finds itself in. Mechanical testing and characterization of the structure of the shell as a function of hydration level, at several length scales, from the micro- to the molecular level, revealed that these shells conform to a hierarchical, non-uniform construction, wherein water absorption within distinct environments facilitates structural adaptation to a changing environment. The discovered design motifs and modifications thereof upon water absorption, underpinning the properties of this natural composite material, will help material scientists to design and synthesize novel stimuli-response materials that are as tough and adaptable as these brachiopod shells.

## Methods

### Materials

Brachiopod *D. tenuis s*hells were collected in Swakopmind, Namibia by Sir Alwyn Williams. The soft tissue was removed, and shells were stored in the air.

### Electron microscopy (EM)

SEM was performed either on a Quanta 650 FEG SEM or on a Zeiss Crossbeam 550 cryoFIB-SEM. HAADF TEM was performed on a Thermofisher Scientific Scios Dual Beam FIB-SEM.

### Sample preparation for electron microscopy

Shell fragments were incubated in MilliQ water overnight at room temperature, followed by fixation in 4% formaldehyde for 4 h. The specimens were then gradually dehydrated in ethanol following a dilution series (50%, 70%, 96%, and 100% ethanol), followed by critical point drying using a Polaron critical point dryer. Subsequently, the shells were embedded in resin, polished, and coated with carbon. To check that the sample preparation procedure does not introduce artifacts such as local sample shrinkage, control experiments on non-treated samples were performed. A comparison between dry shells in their native state and after sample preparation showed no discernible differences. Further, electron microscopy observations, where possible, are cross-validated and confirmed by cryo-PXCT. Moreover, both dry and hydrated samples underwent the same sample preparation, i.e., samples would be largely equally affected by potential preparation artifacts.

### Thermogravimetric analysis (TGA)

TGA was performed using a Thermal Analysis SDT Q600 instrument. A 5–10 mg of ground shell, were subjected to a heating rate of 10 °C/min under nitrogen.

### Fourier transform infrared spectroscopy (FT-IR)

FT-IR spectroscopy measurements were performed using an FTIR Nicolet iS10. Approximately, 2 wt% of ground shell fragments were mixed with KBr and pressed into a transparent disk^48^. Data were acquired between 4000–400 cm^−1^ with a spectral resolution of 4 cm^−1^.

### Differential scanning calorimetry

DSC measurements were performed using a Thermal Analysis SDT Q600 instrument. A 2–5 mg of ground shell was analyzed. Samples were heated up to 240 °C with a rate of 10 °C/min. β-chitin extracted from *D. tenuis* shells was used as a standard. Chitin extraction was done by decalcifying 10–15 mg of shells in 0.55 M HCl twice for 30 min, then once for 1 h at room temperature. The remaining organic material was then incubated in 0.3 M NaOH at 80 °C under reflux for 1 h. The extracted chitin was dried at 80 °C for 1 h^49^.

### Powder X-ray diffraction (PXRD)

Diffraction patterns were collected using an X′Celerator detector fitted on a PANalytical X′Pert Pro diffractometer, using Cu-Kα radiation generated at 40 kV and 40 mA. Data were collected within the 2*θ* range from 5° to 70° with a step size of 0.02° and a counting time of 1200 s. Fixed anti-scatter and divergence slits of 1/16° were used with a 10 mm beam mask.

### Small-angle X-ray scattering (SAXS)

Monochromatic radiation with a wavelength of *λ* = 1.54 Å was produced by a rotating Cu anode (MicroMax 007HF). Scattering patterns were acquired using a Dectris PILATUS 300 K, with a pixel size of 172 μm, placed at a different sample to detector distances between 0.5 and 1.6 m for each dataset. The obtained 2D SAXS patterns were azimuthally integrated, normalized with respect to the incident beam intensity and acquisition time, and then merged to construct a single 1D intensity profile *I*(*q*) vs. *q* covering an effective scattering vector range of 0.0035 to 1 Å^−^^1^. For each sample, at least three SAXS datasets were collected across the shell width. Two representative intensity profiles, i.e., one per sample, are shown in Supplementary Fig. 8.

### Wide-angle X-ray scattering (WAXS)

WAXS data were collected at the 11-BM Complex Materials Scattering (CMS) beamline at National Synchrotron Light Source II (NSLS-II), Brookhaven National Laboratory. Data were collected at 13.5 keV with a beam footprint on the sample of 0.2 × 0.2 mm. The point acquisition time was 10 s. WAXS patterns were recorded with a Pilatus800k placed 0.26 m downstream of the sample. The obtained 2D WAXS patterns were azimuthally integrated, normalized with respect to the incident beam intensity and acquisition time. The resulting 1D intensity profiles are shown in Supplementary Fig. 10.

### Solid-state nuclear magnetic resonance spectroscopy

All experiments were carried out on shells that had been stored at −80 °C, packed into inserts for 4 mm zirconia rotors in a Bruker double-resonance MAS probe on a Bruker AVANCE II 400 MHz wide-bore spectrometer. CP-MAS: MAS frequency 10 kHz, ^1^H 90° pulse 2.5 μs, contact time of 2.5 ms with a ramped pulse on ^1^H and square pulse on ^13^C 70 kHz spin lock field strength, 100 kHz field strength SPINAL64 decoupling during acquisition with 4.4 μs pulses, recycle delay 2 s. Heteronuclear correlation (Hetcor) spectra were recorded at 400 MHz, 10 kHz MAS and 290 K. NMR parameters were: 1H 90° pulse length and decoupling 86 kHz, 1H contact pulse 54 kHz, 31 P contact pulse 44 kHz, 200 µs contact time. Lee–Goldburg (LG) RF field was set to 50 kHz, with an offset for proton evolution under LG of −2000 Hz.

### Depth-dependent dynamic nanoindentation

To prepare the physical cross-sections, the two large shell surfaces were first covered with a thin layer of plasticine (about 1 mm in thickness) before the sample was embedded in epoxy resin and cut to size using a rotating diamond blade. The exposed cross-sections were polished with silicon carbide sandpaper of two decreasing grit sizes (P600 and P1220) and finally using alumina colloidal suspensions with grain sizes of 3-1 and 0.05 microns. Finally, the plasticine was removed with the help of a thin curved dissecting needle generating two cavities (Supplementary Fig. 11). These cavities were filled with double-distilled water for the measurements in hydrated conditions. The same sample was then used for the measurements in dry conditions upon water removal and overnight air drying.

Depth-dependent mechanical properties of dry and fully hydrated shell sections were measured with a nanoindentation tester (model NHT-TTX by CSM Instruments) equipped with a Berkovich diamond tip. The instrument was operated in continuous stiffness mode up to a maximum applied load of 30 mN. During the 60 s loading phase, the oscillatory force modulation had an amplitude equal to 10% of the current force value and a frequency of 5 Hz, while the unloading phase was carried out linearly in 15 s. The instrumented values of the elastic Young’s modulus *E*_IT_ and hardness *H*_IT_ were determined as a function of the indentation depth by Oliver–Pharr dynamic analysis^50^ of the loading phase. The mechanical properties of the tested shell samples were completely reversible in response to the applied hydration-drying cycle.

### Ptychographic X-ray computed tomography (PXCT)

*Sample preparation for PXCT.* Brachiopod shells were first mechanically fractured and cut into mm-sized pieces. Pieces from adjacent areas taken from the environment facing side along the shell width were then glued onto individual custom-build tomography pins^51^. The epoxy was pre-cured and only applied to the top of the tomography pin to avoid sample contamination. The sample-loaded pins were mounted on a custom-built micro-lath and milled under cryogenic conditions^27^. The resulting cylindrical pillars had a diameter of ~20–40 microns and a sample height of ~50–80 microns. The prepared pillars were then either vacuum dried or incubated in desiccators containing salt solutions to create an atmosphere of 70% relative humidity or 100% relative humidity for 36 h^28,29^. The pillars were following frozen using liquid nitrogen to lock the set hydration level in place for the duration of the PXCT measurement. No signs of crystalline ice on the surface of prepared pillars were recorded.

*PXCT setup and data acquisition*. PXCT experiments were carried out at the cSAXS beamline of the SLS. The photon energy was 6.2 keV. The horizontal aperture of slits located 22 m upstream of the sample was set to 20 μm in width to coherently illuminate a Fresnel zone plate, the latter being 220 μm in diameter with an outermost zone width of 60 nm^52^. Coherent diffraction patterns were acquired using a 500k Eiger detector^53^ with a 75 μm pixel size, 7.284 m downstream of the sample. A flight tube was positioned between sample and detector to reduce air scattering and absorption. Measurements were carried out using the positioning instrumentation described in Holler et al.^54,55^. The samples were imaged in an in-vacuum version of this setup at a temperature of −180 °C in a vacuum. Sampling positions were set using a Fermat spiral scanning grid^56^ with an average step size of 2 μm. Tomography projections were acquired using a binary acquisition strategy as described by Kaestner et al.^57^ with two nests of projections. Around 600–1200 projections were acquired depending on the sample diameter. Each projection was obtained by a ptychographic scan of ~400–800 to diffraction patterns, each with an exposure time of 0.1 s.

*Ptychographic image and tomogram reconstruction*. From each diffraction pattern, a region of 512 × 512 pixels was used in the ptychographic reconstruction of acquired projections. The resulting pixel size is (38.8 nm)^2^. Reconstructions were obtained with 300 iterations of the difference map algorithm^58^ followed by 300 iterations of maximum likelihood refinement using 2 probes modes^59,60^. Reconstructions were performed using the PtychoShelves package^61^. Prior to tomography reconstructions, the complex-valued projections were aligned and processed as described in Guizar-Sicairos et al.^62^. Horizontal alignment was ensured based on tomographic consistency^63^. Tomographic reconstruction of phase projections was performed using a modified filtered back-projection algorithm (FBP)^62^. To mitigate noise in the reconstruction, a Hanning filter was used. The tomograms provide the 3D distribution of the refractive index decrement, *δ*(**r**), and electron density away from sample relevant absorption edges as in the present case^24,25^.

*PXCT dose estimation*. The X-ray dose imparted to a shell sample during tomogram acquisition was estimated to be on the order of 10^6^ to 10^7^ Gy. The estimated dose is based on the average area flux density of each scan and the assumed mass density of the specimen^64^. Here, the specimen was assumed to consist of hydroxyapatite and chitin.

*Estimation of spatial resolution*. The half-period spatial resolution of ptychographic tomograms was estimated by Fourier shell correlation (FSC)^65^. The full dataset of angular projections used for the tomographic reconstructions was divided in half, and two independent tomograms with double angular spacing were reconstructed independently. Then, the correlation between these two tomograms in the Fourier domain was calculated and the resolution was estimated based on the intersection with a set threshold. The threshold criteria for the FSC was the ½ bit criteria^65^. FSC line plots of are shown in, Supplementary Fig. 6.

*Tomogram analysis*. Owed to the superior spatial-resolution, the analysis focused on the retrieved phase respectively electron density tomograms^30^. To exclude any potential sample preparation artifacts we extracted sub-volumes (Fig. 3a) from the center of the imaged volume. Figure 3e, electron density line profiles normal to the laminae structure were obtained by calculating the radially averaged electron density of the identifiable layers. The average layer thickness was calculated using a parallel plate model. Overall sample composition and hydration were based on linear combination fitting using the theoretical electron density values of known shell components as well as the measured electron densities of the fully dry shell as reference points. Equally, component matching was achieved by comparing calculated electron densities of known shell components, i.e., francolite, organic matrix (approximated using molecular weight and density of chitin), and water/ice, with the measured electron densities of manually isolated, i.e., visual pure, components in the tomogram where possible. Supplementary Fig. 7 shows local variations in hydration level for the fully hydrated sample calculated from the respective electron density tomogram using the average electron density of the dry sample and the electron density of amorphous ice as reference values. The volume percentages obtained were converted to water weight percent using tabulated density values of shell components. The resulting hydration tomogram was threshold segmented for visualization purposes and to determine the swelling degree of structurally coherent layers as a function of hydration level following using thickness analysis^26,66^.

## Supplementary information

Supplementary Information

Description of Additional Supplementary Files

Supplementary Movie 1

Supplementary Movie 2

Supplementary Movie 3

Supplementary Movie 4

Supplementary Movie 5

Supplementary Movie 6

## Data Availability

The electron microscopy and X-ray computed ptychography generated in this study can be retrieved from the University of Edinburgh DataShare, 10.7488/ds/3056. The remaining data that support the findings reported in this study are available within the paper and its supplementary information files.

## References

[1] Lowenstam, H. A. & Weiner, S. *On Biomineralization*. (Oxford University Press, 1989).

[2] Meldrum FC, Colfen H (2008). Controlling mineral morphologies and structures in biological and synthetic systems. Chem. Rev..

[3] Dunlop JWC, Fratzl P (2010). Biological composites. Annu. Rev. Mater. Res..

[4] Wegst UGK, Bai H, Saiz E, Tomsia AP, Ritchie RO (2015). Bioinspired structural materials. Nat. Mater..

[5] Fratzl P, Kolednik O, Fischer FD, Dean MN (2016). The mechanics of tessellations—bioinspired strategies for fracture resistance. Chem. Soc. Rev..

[6] Smith BL (1999). Molecular mechanistic origin of the toughness of natural adhesives, fibres and composites. Nature.

[7] Aizenberg J (2005). Skeleton of Euplectella sp.: structural hierarchy from the nanoscale to the macroscale. Science.

[8] Weiner S, Wagner HD (1998). The material bone: structure mechanical function relations. Annu. Rev. Mater. Res..

[9] Dunlop JWC, Hartmann MA, Brechet YJ, Fratzl P, Weinkamer R (2009). New suggestions for the mechanical control of bone remodeling. Calcif. Tissue Int..

[10] Frost HM (1987). Bone “mass” and the “mechanostat”: a proposal. Anat. Rec..

[11] Williams A, Cusack M, Buckman JO (1998). Chemico-structural phylogeny of the discinoid brachiopod shell. Philos. Trans. R. Soc. B.

[12] Bayerlein B (2016). Inherent role of water in damage tolerance of the prismatic mineral–organic biocomposite in the shell of *Pinna nobilis*. Adv. Funct. Mater..

[13] Peng J, Snyder GJ (2019). A figure of merit for flexibility. Science.

[14] Klocke D, Schmitz H (2011). Water as a major modulator of the mechanical properties of insect cuticle. Acta Biomater..

[15] Paris, O., Hartman, M. A. & Fritz-Popovsi, G. in *Materials Design Inspired by Nature: Function Through Inner Architecture* (eds Peter Fratzl, John W. C. Dunlop, & Richard Weinkamer) (Royal Society of Chemistry, 2013).

[16] Fabritius H-O, Sachs C, Triguero PR, Raabe D (2009). Influence of structural principles on the mechanics of a biological fiber-based composite material with hierarchical organization: the exoskeleton of the lobster Homarus americanus. Adv. Mater..

[17] Ye XL, Liu LZ, Jin HJ (2016). Responsive nanoporous metals: recoverable modulations on strength and shape by watering. Nanotechnology.

[18] Legeros RZ, Pan CM, Suga S, Watabe N (1985). Crystallo-chemical properties of apatite in atremate brachiopod shells. Calcif. Tissue Int..

[19] Agbaje OBA, George SC, Zhang Z, Brock GA, Holmer LE (2020). Characterization of organophosphatic brachiopod shells: spectroscopic assessment of collagen matrix and biomineral components. RSC Adv..

[20] Williams A, Cusack M, Buckman JO, Stachel T (1998). Siliceous tablets in the larval shells of apatitic discinid brachiopods. Science.

[21] Williams A, Luter C, Cusack M (2001). The nature of siliceous mosaics forming the first shell of the brachiopod discinisca. J. Struct. Biol..

[22] Kaya M (2017). On chemistry of γ-chitin. Carbohydr. Polym..

[23] Tõnsuaadu K, Gross KA, Plūduma L, Veiderma M (2012). A review on the thermal stability of calcium apatites. J. Therm. Anal. Calorim..

[24] Diaz A (2012). Quantitative x-ray phase nanotomography. Phys. Rev. B.

[25] Dierolf, M. et al. Ptychographic X-ray computed tomography at the nanoscale. *Nature* http://www.nature.com/nature/journal/v467/n7314/abs/nature09419.html#supplementary-information (2010). 10.1038/nature09419 20864997

[26] Ihli J (2017). A three-dimensional view of structural changes caused by deactivation of fluid catalytic cracking catalysts. Nat. Commun..

[27] Holler, M. *et al*. A lathe system for micrometre-sized cylindrical sample preparation at room and cryogenic temperatures. *J. Synchrotron Radiat*. 10.1107/S1600577519017028 (2020). 10.1107/S1600577519017028 PMC7064112 32153287

[28] Rockland LB (1960). Saturated salt solutions for static control of relative humidity between 5° and 40 °C. Anal. Chem..

[29] Stokes RH, Robinson RA (1949). Standard solutions for humidity control at 25 °C. Ind. Eng. Chem..

[30] Ihli J (2018). Resonant ptychographic tomography facilitates three-dimensional quantitative colocalization of catalyst components and chemical elements. J. Phys. Chem. C.

[31] Ihli J (2020). Ptychographic X-ray tomography reveals additive zoning in nanocomposite single crystals. Chem. Sci..

[32] Saito Y, Kumagai H, Wada M, Kuga S (2002). Thermally reversible hydration of beta-chitin. Biomacromolecules.

[33] Sawada D (2012). Water in crystalline fibers of dihydrate beta-chitin results in unexpected absence of intramolecular hydrogen bonding. PLoS ONE.

[34] Wang Y (2012). The predominant role of collagen in the nucleation, growth, structure and orientation of bone apatite. Nat. Mater..

[35] Jager C, Welzel T, Meyer-Zaika W, Epple M (2006). A solid-state NMR investigation of the structure of nanocrystalline hydroxyapatite. Magn. Reson. Chem..

[36] Yesinowski JP, Eckert H (1987). Hydrogen environments in calcium phosphates: proton MAS NMR at high spinning speeds. J. Am. Chem. Soc..

[37] Merkel C (2009). Mechanical properties of modern calcite- (*Mergerlia truncata*) and phosphate-shelled brachiopods (*Discradisca stella* and *Lingula anatina*) determined by nanoindentation. J. Struct. Biol..

[38] Fratzl P, Gupta HS, Fischer FD, Kolednik O (2007). Hindered crack propagation in materials with periodically varying Young’s modulus - Lessons from biological materials. Adv. Mater..

[39] Vincent JF, Wegst UG (2004). Design and mechanical properties of insect cuticle. Arthropod. Struct. Dev..

[40] Duer MJ, McDougal N, Murray RC (2003). A solid-state NMR study of the structure and molecular mobility of alpha-keratin. Phys. Chem. Chem. Phys..

[41] Guidoni G, Denkmayr J, Schoberl T, Jager I (2006). Nanoindentation in teeth: influence of experimental conditions on local mechanical properties. Philos. Mag..

[42] Grandfield K, Vuong V, Schwarcz HP (2018). Ultrastructure of bone: hierarchical features from nanometer to micrometer scale revealed in focused ion beam sections in the TEM. Calcif. Tissue Int..

[43] McNally EA, Schwarcz HP, Botton GA, Arsenault AL (2012). A model for the ultrastructure of bone based on electron microscopy of ion-milled sections. PLoS ONE.

[44] Reznikov N, Shahar R, Weiner S (2014). Bone hierarchical structure in three dimensions. Acta Biomater..

[45] Weiner S, Traub W (1992). Bone structure: from ångstroms to microns. FASEB J..

[46] Bevelander G, Nakahara H (1969). An electron microscope study of the formation of the nacreous layer in the shell of certain bivalve molluscs. Calcif. Tissue Int..

[47] Sachs C, Fabritius H, Raabe D (2006). Hardness and elastic properties of dehydrated cuticle from the lobster *Homarus americanus* obtained by nanoindentation. J. Mater. Res..

[48] Adamiano A, Fabbri D, Falini G, Giovanna Belcastro M (2013). A complementary approach using analytical pyrolysis to evaluate collagen degradation and mineral fossilisation in archaeological bones: the case study of Vicenne-Campochiaro necropolis (Italy). J. Anal. Appl. Pyrolysis.

[49] Tolaimate A, Desbrieres J, Rhazi M, Alagui A (2003). Contribution to the preparation of chitins and chitosans with controlled physico-chemical properties. Polymer.

[50] Oliver WC, Pharr GM (2004). Measurement of hardness and elastic modulus by instrumented indentation: advances in understanding and refinements to methodology. J. Mater. Res..

[51] Holler M (2017). OMNY PIN—a versatile sample holder for tomographic measurements at room and cryogenic temperatures. Rev. Sci. Instrum..

[52] Gorelick S (2011). High-efficiency Fresnel zone plates for hard X-rays by 100keV e-beam lithography and electroplating. J. Synchrotron Radiat..

[53] Johnson I (2014). Eiger: a single-photon counting x-ray detector. J. Instrum..

[54] Holler M (2014). X-ray ptychographic computed tomography at 16nm isotropic 3D resolution. Sci. Rep..

[55] Holler M, Raabe J (2015). Error motion compensating tracking interferometer for the position measurement of objects with rotational degree of freedom. Opt. Eng..

[56] Huang X (2014). Optimization of overlap uniformness for ptychography. Opt. Express.

[57] Kaestner A, Münch B, Trtik P, Butler L (2011). Spatiotemporal computed tomography of dynamic processes. Opt. Eng..

[58] Thibault P (2008). High-resolution scanning X-ray diffraction microscopy. Science.

[59] Guizar-Sicairos M, Fienup JR (2008). Phase retrieval with transverse translation diversity: a nonlinear optimization approach. Opt. Express.

[60] Thibault P, Guizar-Sicairos M (2012). Maximum-likelihood refinement for coherent diffractive imaging. N. J. Phys..

[61] Wakonig K (2020). PtychoShelves, a versatile high-level framework for high-performance analysis of ptychographic data. J. Appl. Crystallogr..

[62] Guizar-Sicairos M (2011). Phase tomography from x-ray coherent diffractive imaging projections. Opt. Express.

[63] Guizar-Sicairos M (2015). Quantitative interior x-ray nanotomography by a hybrid imaging technique. Optica.

[64] Howells MR (2009). An assessment of the resolution limitation due to radiation-damage in X-ray diffraction microscopy. J. Electron. Spectrosc. Relat. Phenom..

[65] van Heel M, Schatz M (2005). Fourier shell correlation threshold criteria. J. Struct. Biol..

[66] Hildebrand T, Rüegsegger P (1997). A new method for the model-independent assessment of thickness in three-dimensional images. J. Microsc..

[67] Ihli, J. et al. *Mechanical Adaptation of Brachiopod Shells Via Hydration-Induced Structural Changes*. (University of Edinburgh, DataShare) 10.7488/ds/3056 (2021). 10.1038/s41467-021-25613-4 PMC8433230 34508091

[68] Momma K, Izumi F (2011). VESTA 3 for three-dimensional visualization of crystal, volumetric and morphology data. J. Appl. Crystallogr..

[69] Nishiyama Y, Noishiki Y, Wada M (2011). X-ray structure of anhydrous β-chitin at 1 Å resolution. Macromolecules.

