# Supplementary material for: Mechanical adaptation of brachiopod shells via hydration-induced structural changes
Source: Nat Commun. 2021 Sep 10;12:5383. doi: 10.1038/s41467-021-25613-4 (PMC8433230; doi:10.1038/s41467-021-25613-4)
Supplement: Supplementary file 1 — Supplementary Information [file 41467_2021_25613_MOESM1_ESM.docx]

Supplementary Information

**Mechanical Adaptation of Brachiopod Shells a Result of Hydration Induced Structural Changes**

*Johannes Ihli*, Anna S. Schenk, Sabine Rosenfeldt, Klaus Wakonig, Mirko Holler, Giuseppe Falini, Luca Pasquini, Eugénia Delacou, Jim Buckman, Thomas S. Glen, Thomas Kress, Esther H.R. Tsai, David G. Reid, Melinda J. Duer, Maggie Cusack, Fabio Nudelman**

**Supplementary Information**

This Supplementary Information Includes:

- Supplementary Figure 1-11
- Supplementary Table 1
- Supplementary Movies 1-6
- Supplementary References

**
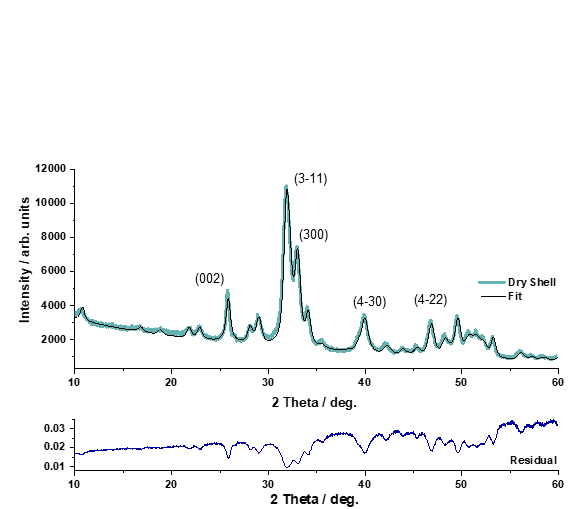
**

**Supplementary Fig. 1. Powder X-ray Diffraction Pattern of a Brachiopod *Discinisca Tenuis* Shell**. One-dimensional diffraction pattern of the powder of a dry brachiopod shell. Data was acquired at 8.04 keV. All reflections can be assigned to fluoroapatite.^1^ In consideration of the rod-shaped bundles and a difference in peak broadening between selected reflections, we utilized an anisotropic size-strain model^2^ during Rietveld refinement^3^. Refinement suggests the average fluoroapatite or francolite nanocrystal to be asymmetric in shape, with a short axis coherence length of 3-6 nm in diameter and a long axis of 14-19 nm in diameter. The corresponding microstrain is suggested to be on the order of 10^-3^ and 10^-4^. Refinements using an anisotropic size-strain model possessed a goodness of fit of ~1.9. Refinement using no such model resulted in a slightly worse fit of ~ 2.2. The suggested coherent domain size in this case is ~16-20 nm with a microstrain of 10^-4^.

**Supplementary Fig. 2**. **Thermogravimetric Analysis of an Atmospherically Dry and Hydrated Brachiopod *Discinisca Tenuis* Shell.** Thermogravimetric analysis of dry shell stored in air (orange) and a hydrated shell created by immersion in H_2_O for 24 hours (grey). Measurements used a continuous heating rate of 10 °C min^-1^ in air. Losses under 40°C where disregarded being ascribable to external surface bound water.

**Supplementary Fig. 3**. **Fourier Transform Infra-Red Spectroscopy of an Atmospherically Dry Brachiopod *Discinisca Tenuis* Shell.** In total, we analysed a dry shell sample at room temperature and after heating to 700 ºC and 900 ºC. To note is the disappearing carbonate band above a temperature of 700 ºC. Further evident is the decomposition of organic matrix components with increasing temperature as evidenced by a reduction in amide associated vibrational modes.


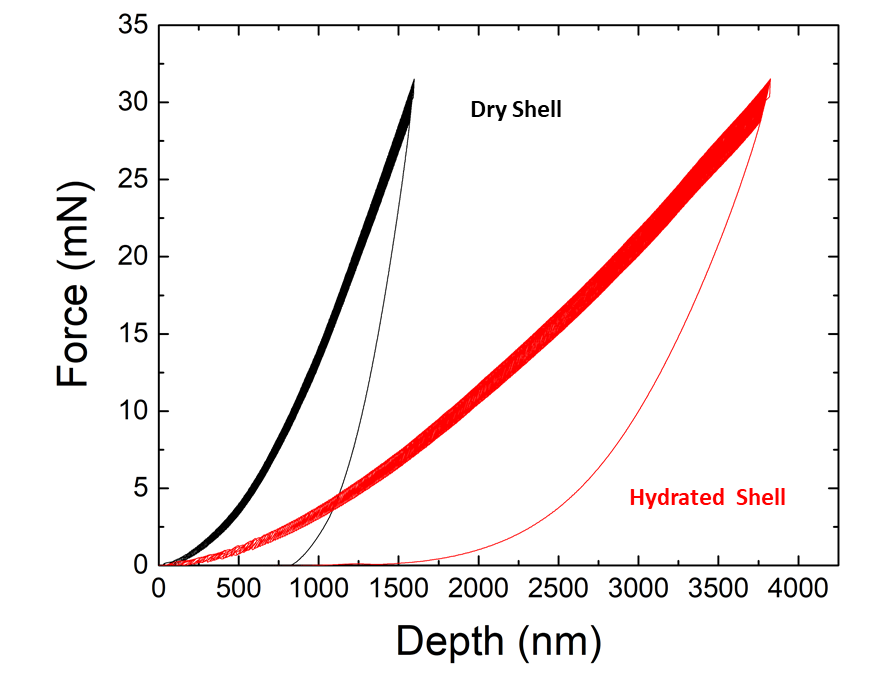


**Supplementary Fig. 4. Typical Force-Indentation Depth Curves of a Dry and Hydrated Brachiopod Discinisca tenuis Shell Sample.** Data was recorded in continuous stiffness mode on both samples up to a maximum force of 30 mN.


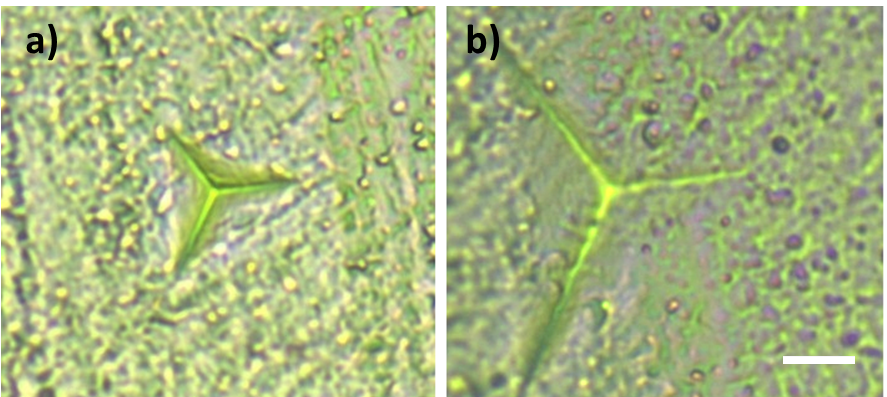


**Supplementary Fig. 5. Residual Imprint After a Dynamic Indentation Measurement on the Dry (a) and Hydrated (b) Shell Sample**. The maximum force was 30 mN in both cases. The scale bar is 5 µm.

**
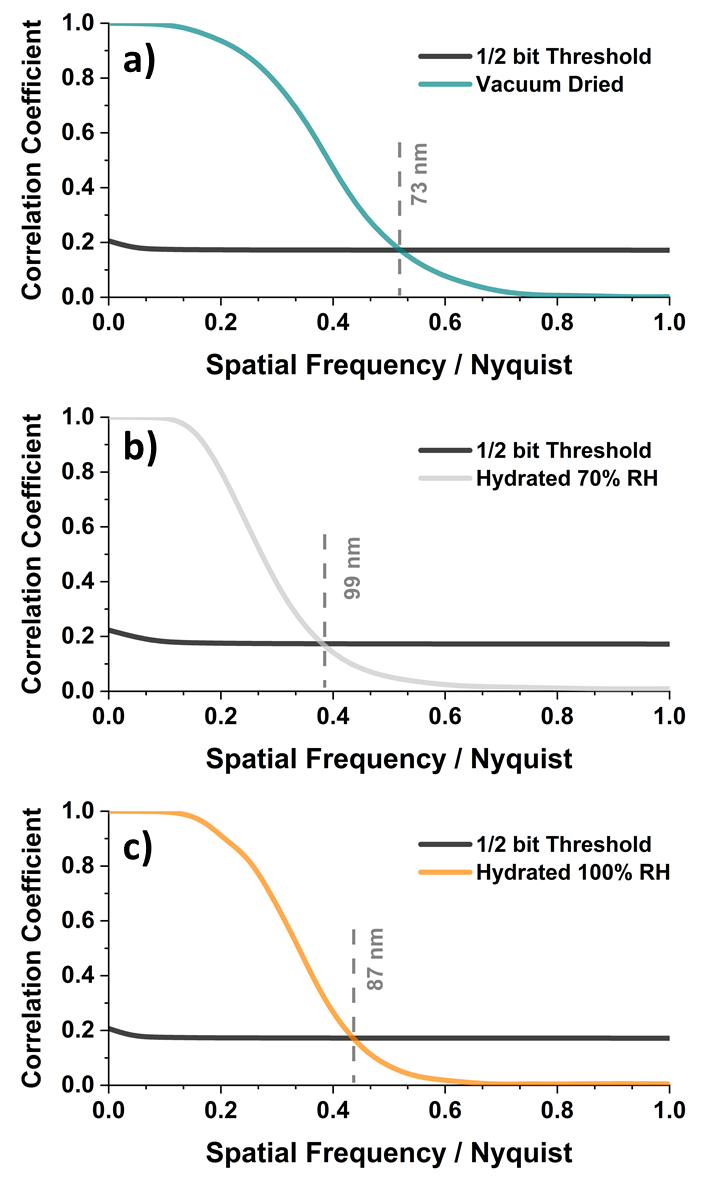
**

**Supplementary Fig. 6. Spatial-Resolution of Ptychographic Tomograms.** Fourier shell correlation (FSC) line plots of the acquired electron density tomograms. The selected threshold for determining the resolution is the ½ bit criterion. The voxel size for all tomograms is (38.8 nm)^3^. The half-period spatial resolution estimate for the (a) dried sample is 73 nm, for the (b) semi-hydrated sample 99 nm and for the (c) fully hydrated sample 87 nm.


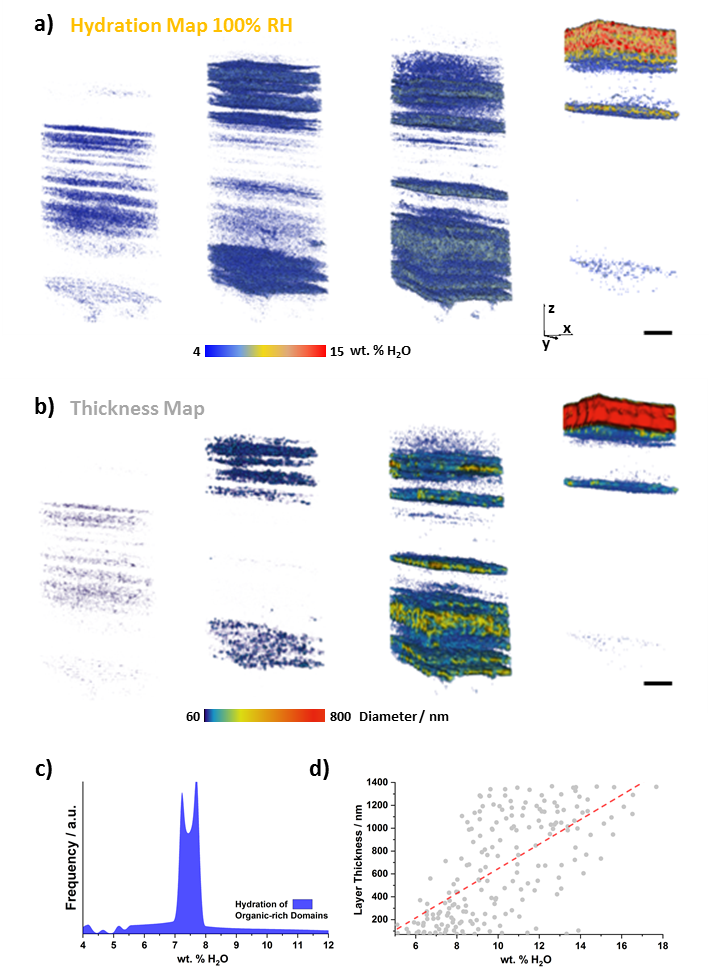


**Supplementary Fig. 7. Local Variation in Shell Hydration and Associated Volume Expansion.** (a) Shown is a series of percent water weight converted volume renderings of the fully hydrated sample first shown in Figure 2d. Presented from left to right are partial volume renderings of voxels possessing, 4-6, 5-7.5, 8.5-10 and 10-15^+^ wt. % H*_2_*O. Common to all panels is a single colour scale ranging from blue to red representative of the approximate water weight on a voxel level. (b) Corresponding thickness maps to evaluate the diameter of the compositionally coherent domains. Common to all thickness maps is a single colour scale ranging from black to red representative of the organic-rich layer thickness or diameter. Smallest spheres considered for thickness map calculations were 76 nm in diameter. Voxel with a water weight of less than 4% were omitted to isolate organic-rich domains. Scale bars are 2 µm. Segmentation and a partial volume display was required, to ease layer visibility and to facilitate thickness map analysis. Segmentation threshold values were histogram guided (c). (d) Scatterplot showing the correlation between the water weight of the organic-rich layer on the voxel level and associated layer thickness. Provided in red is a linear fit, with an overall goodness of fit of 0.65. See Supplementary Movie 6 for an animated presentation of the tomograms.

**
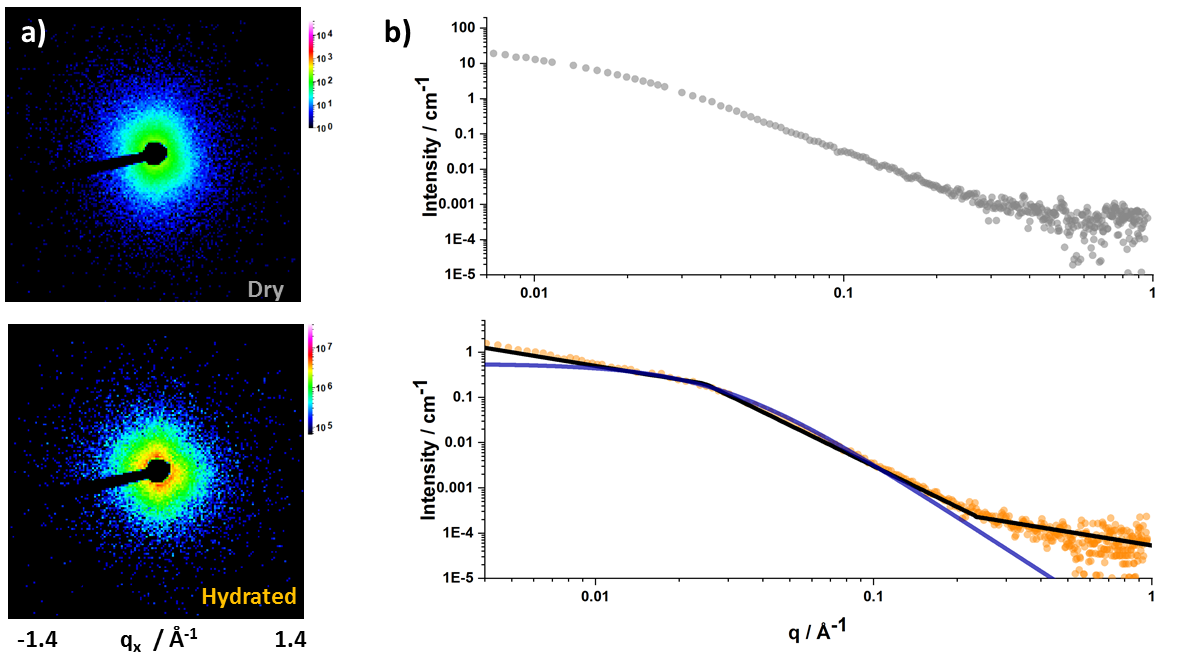
**

**Supplementary Fig. 8. Small-Angle X-ray Scattering of an Atmospherically Dry and a Fully Hydrated Brachiopod *Discinisca Tenuis* Shell**. (a) 2D scattering patterns obtained from an atmospherically dry (top) and fully hydrated shell (bottom). The shell height was aligned perpendicular to the X-ray beam. The 2D SAXS patterns show a substantial anisotropy in the signal most likely originating from organic/inorganic interfaces within the nanocomposite. The maximum expression of this anisotropy reflecting the orientation of anisometric nanostructural motifs is detected in two roughly perpendicular directions, in line with the distorted mineral brickwork structure seen in electron micrographs in Figures 1 and 4 of the main text. In agreement with imaging data the anisotropy in the SAXS signal appears to be slightly more pronounced in the dry sample on average, while the signal is more diffuse and smeared out in the in the fully hydrated sample, where this indicates a higher degree of nanostructural order in the dry state. However, it has to be noted that due to their curved shape the shells are slightly bent with respect to beam axis. Hence, while our approach allows for a qualitative analysis of orientation effects, it does not endow us with the possibility to unequivocally identify the 2D SAXS pattern corresponding to the maximum orientation of the nanostructural motifs in each sample. (b) The corresponding orientation-averaged 1D SAXS intensity profiles of the dry and hydrated sample generally show very similar curve shapes in the considered angular range and are best described by a series of power laws separated by two pronounced kinks at 0.0247 Å^-1^ and 0.23 Å^-1^ (blue curve). Specifically, at low q a power law fit I(q)~q^-1^ for q ≤ 0.0247 Å^-1^ points to cylindrically shaped scattering objects with a radius around 2π/q_cutoff_ = 25 nm.^4^ In the intermediate q range 0.0247 Å^-1^ < q ≤ 0.23 Å^-1^ a power law I(q)~q^-3^ is observed, which may be indicative of fractal-like interfaces, e.g. irregularly delineated pores. ^5^ At large q 0.23 Å^-1^ < q ≤ 1 Å^-1^ the cutoff for the I(q)~q^-1^ power law (which describes the curve comparably well despite the limited statistics in this q range) indicates nanostructural motifs with elongated shape and roughly 2π/q_cutoff_ = 2.7 nm size, which is in good agreement with the PXRD analysis, Supplementary Fig. 1.

Alternatively, a Debye-Anderson-Brumberger (DAB) model^6^ ^6^ (cyan curve) can be used to describe the profiles in the intermediate q-range 0.012 Å^-1^ < q ≤ 0.12 Å^-1^ yielding a correlation length of 3.5 nm, which indicates the average separation between two randomly distributed phases with smooth interfaces. At small q (q ≤ 0.012 Å^-1^) the DAB model does not account for 3-dimensional structures, and thus deviates from the measured data.

**
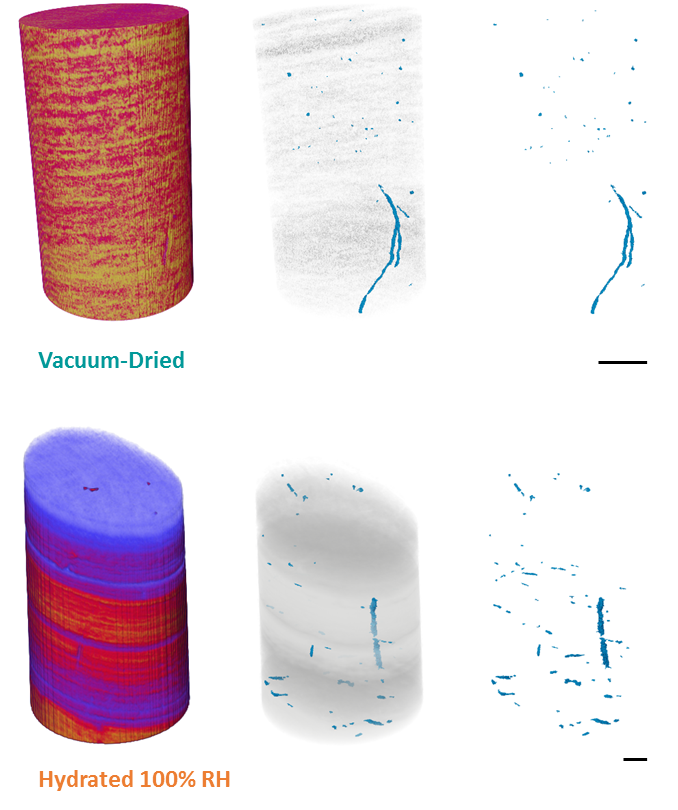
**

**Supplementary Fig. 9. Pore Structure Within a Vacuum-Dried and a Hydrated Brachiopod *Discinisca Tenuis* Shell Sample.** Shown are pore-structures extracted from the electron density tomograms (Figures 3b and d) of two of the imaged sample cylinders. Provided in the top row is the vacuum-dried sample cylinder, in the bottom row the hydrated sample cylinder. Shown in the left column are electron density tomogram volume renderings. These renderings follow a common colour map ranging from white (0.4) to yellow (0.63) representative of electron density values. Shown in the middle column is the location of present and segmented pores (blue) within the sample cylinder highlighted through an overlay with a semi-transparent electron density tomogram outline (grey). Shown in the right column are the identified pores on their own. Pores were extracted though means of machine learning based segmentation.^7^ Scale bars are 2 µm. See also Supplementary Movie 3 and 4.

**
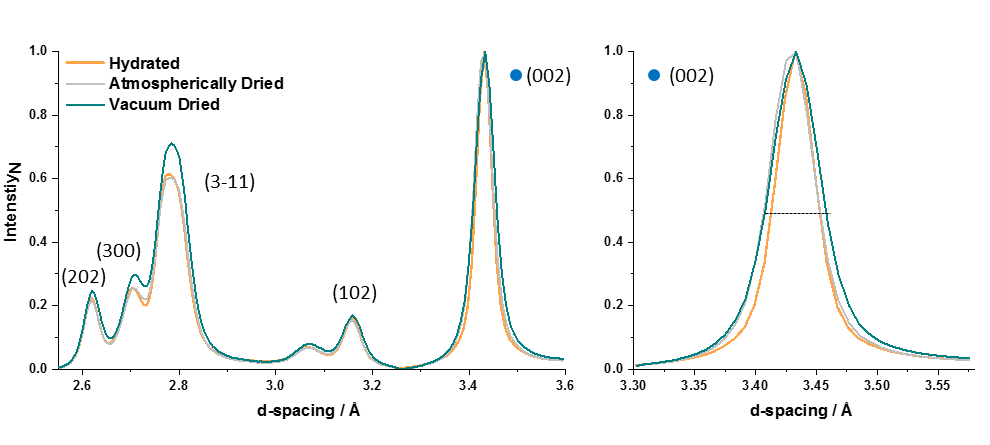
**

**Supplementary Fig. 10. Wide-Angle X-ray Scattering of the Same Brachiopod *Discinisca Tenuis* Shell First Hydrated and Following Atmospheric Drying**. Patterns were acquired at 13.5 keV. The laminae structure of the shell was placed perpendicular to the propagation direction of the illumination. Initially mounted was a hydrated shell sample, obtained after 24 hours of immersion in H_2_O. WAXS patterns were collected directly after mounting of the hydrated shell (orange), following storage in air for 1 hour (grey) and after vacuum drying for 1 hour (turquoise), operations sufficient to dry out the shell, i.e. to revert to its rigid structure. The integrated data was converted from q-space to d-spacing and truncated in the displayed scattering angle to better showcase: (1) the observable decrease in Francolite unit cell dimension as the shell changes from hydrated to dry as evident in the overall shift of Bragg reflection centres to a smaller d-spacings, (2) apparent increase in Bragg reflection peak width upon drying, potentially reflective of an increase in micro and macro strain and (3) an apparent change in the Bragg reflection intensity ratio. Specifically, we refer to a relative increase in the (300) and (3-11) reflections of Francolite as the shell dries. We tentatively assign these changes to a partial reorientation of the rod-shaped mineral bundles. Similar real-space observations were seen in Figure 4. In view of the mounting geometry of the shell for these measurements, we speculate the crystallographic c-axis, representing the short axis of the francolite nanocrystals to be aligned normal to the laminar structure of the shell, in turn, the crystallographic a/b-axis run preferentially parallel to the laminar structure. We are working towards an-depth study that clarifies structural orientation and changes to the francolite crystal structure.

**
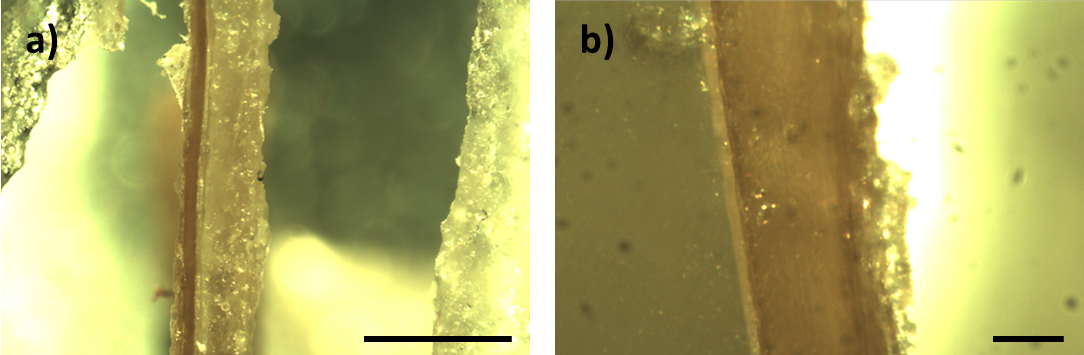
**

**Supplementary Fig. 11**. **Optical Micrographs of a Selected Embedded and Polished Shell Sample Intended for Nanoindentation Measurements.** (a) The sample after embedding in an epoxy resin and after the removal of the plasticine layer. This removal generates two lateral pulls that were filled with water to measure the hydrated shell. (c) Magnified image of the prepared cross section of the shell sample. The shell thickness is about 70 µm. Scale bars are 1 mm (a) and 50 µm (b).

**Supplementary Table 1.**  **Hardness H_IT_ and Young’s modulus E_IT_ of the Same Brachiopod *Discinisca Tenuis* Shell Atmospherically Dry and Fully Hydrated.** Values were determined via depth-resolved nanoindentation. The same shell sample was measured first hydrated and following atmospheric drying. A maximum force of 30 mN was applied in both cases. Values are the average of eight different experiments. The number in parenthesis in the table represents the standard deviation in units of the last digit.

|  | **H_IT_ (MPa)** | **E_IT_ (GPa)** |
| --- | --- | --- |
| **Dry** | 700 (80) | 9.2 (6) |
| **Hydrated** | 156 (24) | 2.4 (2) |

**Supplementary References:**

1. Jahnke RA. The synthesis and solubility of carbonate fluorapatite. *Am. J. Sci.* 1984, **284:** 58-78.

2. Popa N. The (hkl) Dependence of Diffraction-Line Broadening Caused by Strain and Size for all Laue Groups in Rietveld Refinement. *J. Appl. Crystallogr.* 1998, **31**(2)**:** 176-180.

3. McCusker LB, Von Dreele RB, Cox DE, Louer D, Scardi P. Rietveld refinement guidelines. *J. Appl. Crystallogr.* 1999, **32**(1)**:** 36-50.

4. Glatter O, Kratky O. *Small angle x-ray scattering*. London: Academic Press Inc. Ltd: London, 1982.

5. Bale HD, Schmidt PW. Small-Angle X-Ray-Scattering Investigation of Submicroscopic Porosity with Fractal Properties. *Phys. Rev. Lett.* 1984, **53**(6)**:** 596-599.

6. Debye P, Jr. HRA, Brumberger H. Scattering by an Inhomogeneous Solid. II. The Correlation Function and Its Application. *J. Appl. Phys.* 1957, **28**(6)**:** 679-683.

7. Ignacio Arganda-Carreras VK, Curtis Rueden, Kevin W. Eliceiri, Johannes Schindelin, Albert Cardona, H. Sebastian Seung. Trainable Weka Segmentation: a machine learning tool for microscopy pixel classification. *Bioinformatics* 2017, **180**.

8. Cho G, Wu Y, Ackerman JL. Detection of hydroxyl ions in bone mineral by solid-state NMR spectroscopy. *Science* 2003, **300**(5622)**:** 1123-1127.
