# Supplementary material for: Mechanical adaptation of brachiopod shells via hydration-induced structural changes
Source: Nat Commun. 2021 Sep 10;12:5383. doi: 10.1038/s41467-021-25613-4 (PMC8433230; doi:10.1038/s41467-021-25613-4)
Supplement: Supplementary file 2 — Description of Additional Supplementary Files [file 41467_2021_25613_MOESM2_ESM.docx]

**Description of Additional Supplementary Files**

**Supplementary Movie 1:** Video demonstrating the malleability difference between a brachiopod Discinisca tenuis shell fragment in its hydrated state (1h storage in H_2_O) and after drying at 25ºC under nitrogen for 1 hour. The same shell fragment is shown. A blue square is approximately 1 cm in diameter.

**Supplementary Movie 2:** Video example showing the fabrication of a ptychographic tomography sample using a liquid nitrogen cooled micro-lath.

**Supplementary Movie 3:** Volume rendering of the ptychographic tomogram of the vacuum-dried shell. Shown are volume reconstructions of and cut slices through the retrieved electron density tomograms. The beam direction is along the y axis, with the z axis vertical, while the sample holder is placed normal to the beam direction (y).

**Supplementary Movie 4:** Volume rendering of the ptychographic tomogram of the shell exposed to 70 % relative humidity. Shown are volume reconstructions of and cut slices through the retrieved electron density tomograms. The beam direction is along the y axis, with the z axis vertical, while the sample holder is placed normal to the beam direction (y).

**Supplementary Movie 5:** Volume rendering of the ptychographic tomogram of the shell exposed to 100 % relative humidity. Shown are volume reconstructions of and cut slices through the retrieved electron density tomograms. The beam direction is along the y axis, with the z axis vertical, while the sample holder is placed normal to the beam direction (y).

**Supplementary Movie 6:** Partial volume rendering of and orthoslices through the fully hydrated sample tomogram. Shown are hydration and thickness map analysis.
